# Data-Driven Serendipitous Discovery of a Self-Organized Nanostructure for Enhanced Pinning in Superconducting Films

**DOI:** 10.1021/acsnano.6c05455

**Published:** 2026-08-03

**Authors:** Tomoya Horide, Shunta Ito, Ataru Ichinose, Yutaka Yoshida

**Affiliations:** † Department of Electrical Engineering, 12965 Nagoya University, Furo-cho, Chikusa, Nagoya 464-8603, Japan; ‡ Grid Innovation Research Laboratory, 133656 Central Research Institute of Electric Power Industry, Yokosuka, Kanagawa 240-0196, Japan

**Keywords:** self-organization, high temperature superconductor, film, nanocomposite, machine learning, Gaussian process regression, serendipity

## Abstract

In nanocomposite-coated conductors of superconducting YBa_2_Cu_3_O_7_, an improvement of the critical current density is required for high-field applications. After two decades of development, more effective nanostructures are still required to further enhance the vortex pinning. While researcher-dependent empirical approaches are less effective for complex phenomena, serendipity can lead to discoveries beyond established theories, existing knowledge, and trends. Serendipity has been considered an uncontrollable, lucky, or accidental event, and researchers often overlook such events. We propose the concept of serendipity engineering. Here, we regard fluctuation-induced anomalies in experiments as a potential driver for serendipity and detect such fluctuation-induced anomalies using an anomaly score defined by log likelihood. We implemented data-driven serendipity detection for the discovery of structures and physical mechanisms. By applying this concept to nanoscale self-organization of the YBa_2_Cu_3_O_7_ nanocomposite multilayer, we improved the critical current density. Instead of forming the designed multilayer structure, spontaneous structural reconstruction occurred across several layer units, resulting in weak or random correlations between nanorods. Spacer layers between nanorods, which enable flexible vortex configurations, play a key role in enhancing the vortex pinning in magnetic fields. Weakly correlated nanorods represent an unexpected optimal pinning structure, leading to pseudo-high-density vortex pinning and a 2.9-fold enhancement in the critical current density. This study demonstrates the potential of serendipity engineering for discovery at the nanoscale self-organization.

## Graphical abstract

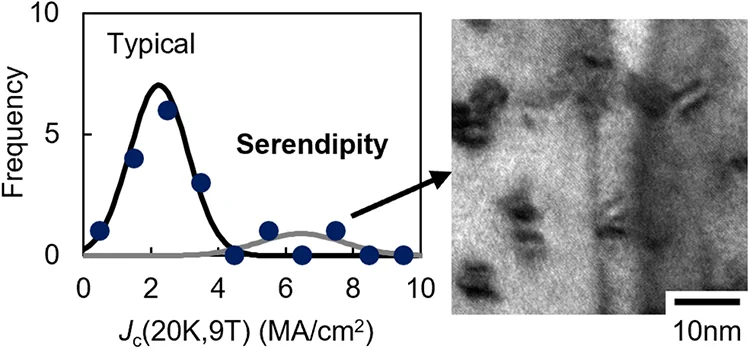

## Introduction

YBa_2_Cu_3_O_7_ (YBCO)-coated conductors with kilometer-scale lengths are industrially produced for applications in fusion systems, high-resolution nuclear magnetic resonance, high-field magnets, and particle accelerators.^1–3^ YBCO-coated conductors are fabricated on ion-beam-assisted deposition (IBAD) templates. The vortex configuration and dynamics governed by the pinning potential, line tension, and vortex–vortex interactions determine the critical current density (*J*_c_).^4,5^ For applications, enhancing the vortex pinning and *J*_c_ requires nanoscale structural engineering.^6^ Self-organized nanocomposite structures containing nanorods^7–11^ and nanoparticles^12^ act as highly effective pinning centers, and control of vortex pinning has been extended to Fe-based superconductors.^13^ Although nanorods and nanoparticles have been extensively studied, more effective nanostructures are still required to further enhance the vortex pinning. A variety of advanced control strategies have been investigated.^14–21^ An intermediate configuration between nanorods and nanoparticles is promising for controlling the vortex configuration and dynamics; however, its design remains challenging due to structural complexity and processing constraints.

Researchers propose hypotheses on the basis of their experience, knowledge, theory, simulations, and experiments. New phenomena, materials, and structures are then discovered through numerous experimental trials and errors guided by these hypotheses. However, in the case of vortex pinning, the conventional approach may be becoming less effective for making discoveries as two decades of research have already explored accessible hypotheses. In addition to traditional hypothesis-driven methods, machine learning has recently emerged as a powerful tool for materials discovery and optimization.^22–24^ However, it is difficult for machine learning to extrapolate beyond the known data.

Serendipity will play a key role in enabling scientific discoveries and breakthroughs.^25^ Figure 1 shows the concept of serendipity engineering and serendipitous discoveries in the material fabrication process. Although optimization is performed within a given parameter space, unintended fluctuations can occur in experiments, causing the system to deviate from the designed regions. Such strong fluctuations may arise from several unintended experimental factors, including operator-dependent variability, instrumental effects, and the intrinsic stochasticity of the system. Researchers can take advantage of unexpected events in an undesigned parameter space, namely, serendipity, only when they are able to detect anomalies.

**Figure 1:**
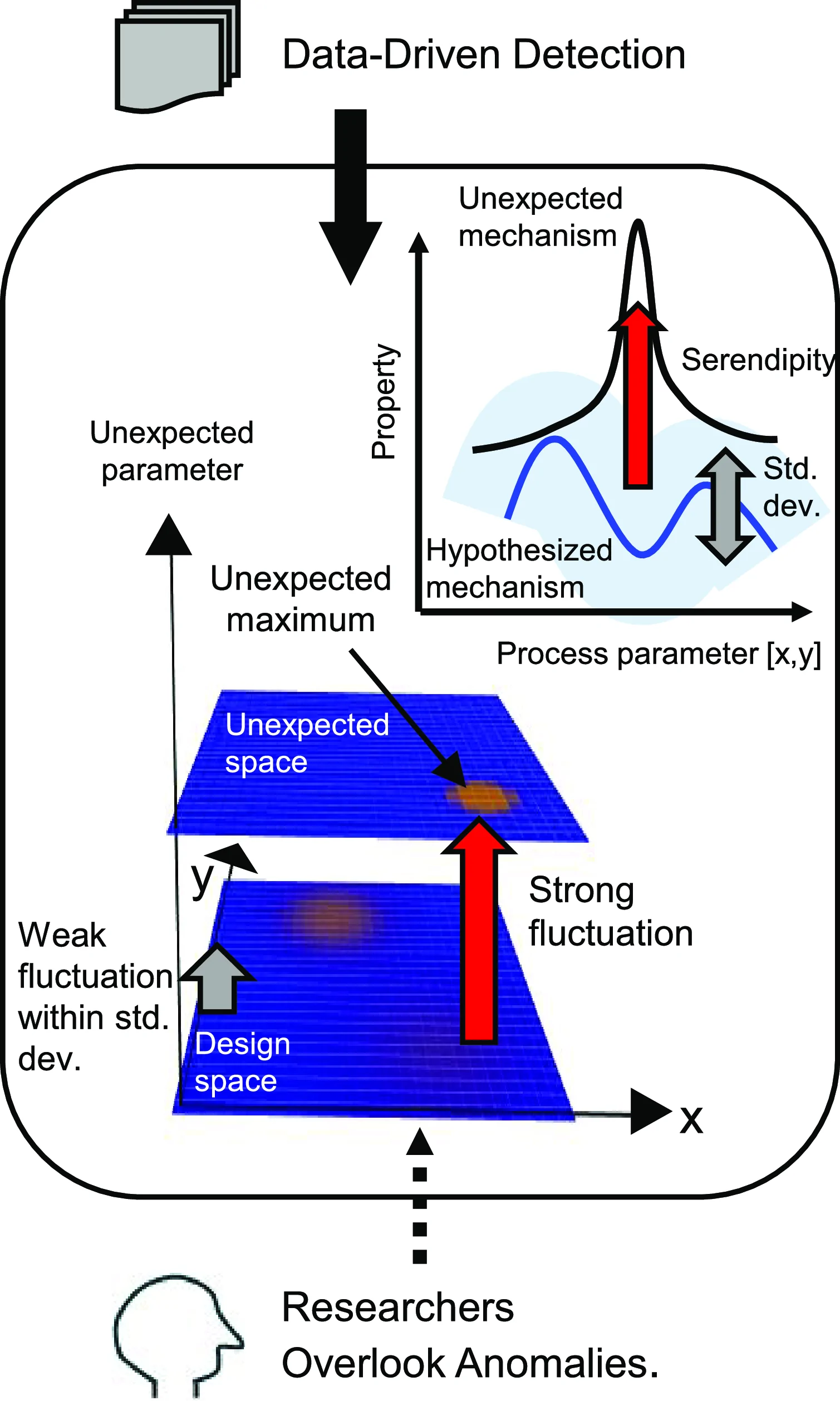
Schematic illustration of the concept of this study. Unexpected maxima in properties may exist outside the optimization parameter space. Strong fluctuations in experiments may reach an unexpected parameter space and unexpected maxima. Although researchers often overlook anomalous events, a data-driven method detects them independently of researchers’ intuition and experience.

Lee et al. have discussed serendipity engineering.^26^ While their discussion was conceptual and focused on biology and medicine, they discussed a framework comprising the expansion of the observable space, retention of anomalies, establishment of analytical strategies, and cultivation of openness. Although the concept is intuitive, its practical implementation has been challenging. Anomaly detection depends on the intuition and experience of researchers. By preventing anomalies from being overlooked, serendipitous discoveries can be achieved independently of researchers. Reliable detection of strong fluctuations may serve as a potential driver of serendipitous discoveries. Our approach to serendipity, which includes strong fluctuations in experiments, measurements of all samples, GPR-based anomaly analysis, and a detailed investigation of anomalous samples, is consistent with that of their framework. We applied this concept to YBCO films to achieve a serendipitous discovery of a self-organized nanostructure. Through detailed analysis, we clarified the pseudo-high-density pinning structure in YBCO films. This demonstrates the potential strategy of serendipitous discoveries in materials, which enables even robotic systems to make serendipitous discoveries.

## Results

### Observation of Anomaly

YBa_2_Cu_3_O_7_+BaHfO_3_/YBa_2_Cu_3_O_7_ (YBCO+BHO/YBCO) multilayer films^27–30^ were fabricated based on a Bayesian optimization framework. The corresponding data sets are shown in Table S1. Figure 2A shows the result of conventional Bayesian optimization for *J*_c_(20 K, 9 T) without anomaly exclusion. Since YBCO tapes are used at 20 K under high magnetic fields for fusion applications, *J*_c_(20 K, 9 T) in YBCO films on IBAD templates is discussed for applications in this study. In this Bayesian optimization, *J*_c_(20 K, 9 T) exhibited an overall increasing trend with the sample number, despite the occurrence of abnormally high *J*_c_ values. To perform Bayesian optimization, Gaussian process regression (GPR) was constructed.^31–34^ As shown in Figure 1, weak fluctuations can be treated as statistical errors within a single GPR model. In contrast, when strong fluctuations lead to anomalous events, they should be treated separately to construct an accurate model. To demonstrate this in a real experimental system, the influence of anomalies on the accuracy of the GPR model is discussed.

**Figure 2:**
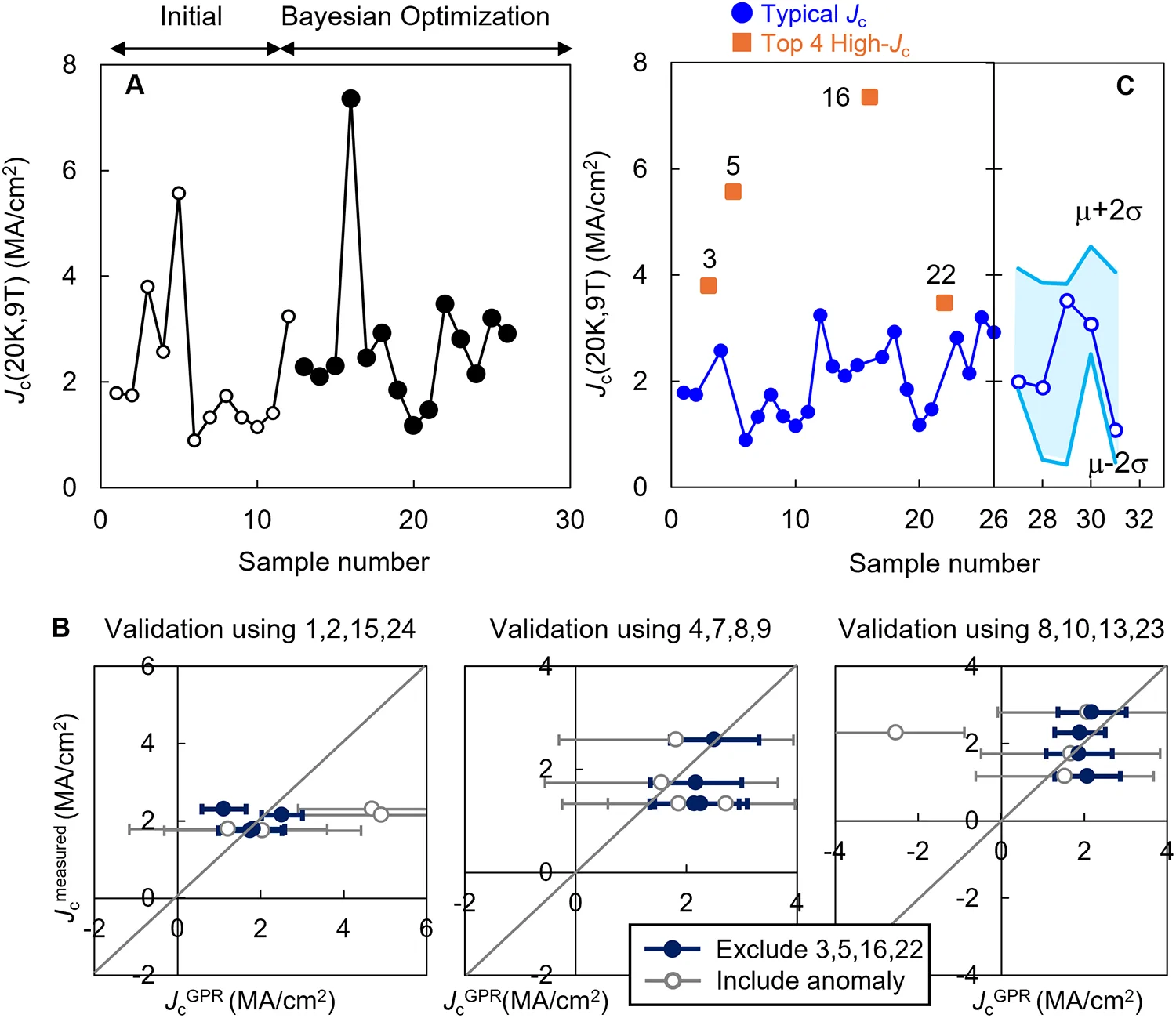
(A) Variation of *J*_c_(20 K,9 T) with the sample number in the conventional Bayesian optimization. (B) Validation of Gaussian process regression. The measured *J*_c_ (*J*_c_^measured^) and GPR-predicted *J*_c_ (*J*_c_^GPR^) are compared. Dark-blue points and error bars show the GPR results for the data set excluding the top four *J*_c_ data points (Samples 3, 5, 16, and 22), whereas gray points and error bars show the results including them. The validation data sets are Samples 1, 2, 15, 24 (left panel), Samples 4, 7, 8, 9 (center panel), and Samples 8, 10, 13, 23 (right panel). (C) Variation of *J*_c_(20 K, 9 T) with the sample number. For Samples 13–26, the *J*_c_ model was constructed using conventional Bayesian optimization with the anomalous data included. After Sample 27, the GPR model was constructed excluding the top four highest *J*_c_ samples.

Figure 2B shows the measured *J*_c_ (*J*_c_^measured^) and GPR-predicted *J*_c_ (*J*_c_^GPR^) for data sets including and excluding anomalies (Samples 3, 5, 16, and 22). When the GPR model is constructed from the data set including anomalies, four data points are used for validation and the remaining 22 data points are used for model construction. In this case, the model fails to predict some of the measured *J*_c_ values even when taking the large standard deviation into account. Furthermore, the confidence interval of the GPR model trained on the data set containing anomalies includes negative values. Since the confidence interval in GPR is mathematically determined by the characteristics of the training data set, the prediction performance is strongly influenced by the data set properties. The appearance of negative values indicates that the GPR model trained with anomalous data fails to accurately predict *J*_c_, thereby demonstrating the necessity for anomaly exclusion.

It is reasonable to construct a GPR model by using data without anomalies. The top four highest *J*_c_ values (Samples 3, 5, 16, and 22) were excluded as anomalies, and an additional four randomly selected data points were reserved for validation. After removing the anomalies and the data for validation, the remaining data set consisting of 18 samples was used for model construction. When the anomalies were excluded, the predicted *J*_c_ values consistently agreed with the measured *J*_c_ values within the standard deviation, regardless of which data points were removed. This result demonstrates that the GPR model can predict measured *J*_c_ when anomalous data points are excluded. Here, the top four highest *J*_c_ values were regarded as anomalies, which is a tentative selection. Even if the exclusion includes some normal data in addition to anomalies, the model accuracy is not significantly affected, although the reduced amount of data may have a slight impact on performance. Therefore, tentative selection is sufficient at this stage.

Bayesian optimization was performed with a GPR model excluding the four data points (Samples 3, 5, 16, and 22) with the highest *J*_c_ values. The corresponding Bayesian optimization results for Samples 27–31 are shown in Figure 2C. The measured values fell within μ ± 2σ, indicating that the GPR model was successfully constructed. Excluding anomalies from the initial data improved the accuracy of the subsequent optimization. In conventional data-driven approaches, failures or anomalies are typically undesirable and are generally regarded as negative phenomena that need to be addressed because a single predictive model is typically assumed.^35,36^ In contrast, in this study, anomalies are positively regarded as potential drivers of serendipitous discovery. For this purpose, accurate identification of anomalies is required.

### Anomaly Detection Based on Data

Figure 3A shows a *J*_c_(20 K, 9 T) histogram for the YBCO+BHO/YBCO at the neighboring condition window of Sample 16. Statistical analysis was performed using a subset of the samples shown in Figure 2 and additional 16-series samples (16–1, 16–2, etc.), which have identical nominal multilayer parameters. The bin width is 1, and the total number of samples is 16. Detailed sample information is shown in Table S2. The samples have a ratio = 0.8, repetition = 26–34 (YBCO+BHO layer thickness = 5–7 nm, YBCO spacer layer thickness = 1–2 nm), BHO volume fraction of 1–2%. Thus, the conditions vary slightly within this window. The mean value and standard deviation for the low-*J*_c_ population are 2.2 MA/cm^2^ and 0.87 MA/cm^2^, respectively. A bimodal structure is observed. (μ = 6.5, σ = 1.3) and (μ = 2.2, σ = 0.87) correspond to Samples 5 and 16, and the remaining samples, respectively. Since (μ = 6.5, σ = 1.3) is estimated from the two data points, statistical reliability is insufficient. However, the high *J*_c_ values of Samples 5 and 16 are evident and cannot be explained by the distribution of the low-*J*_c_ population.

**Figure 3:**
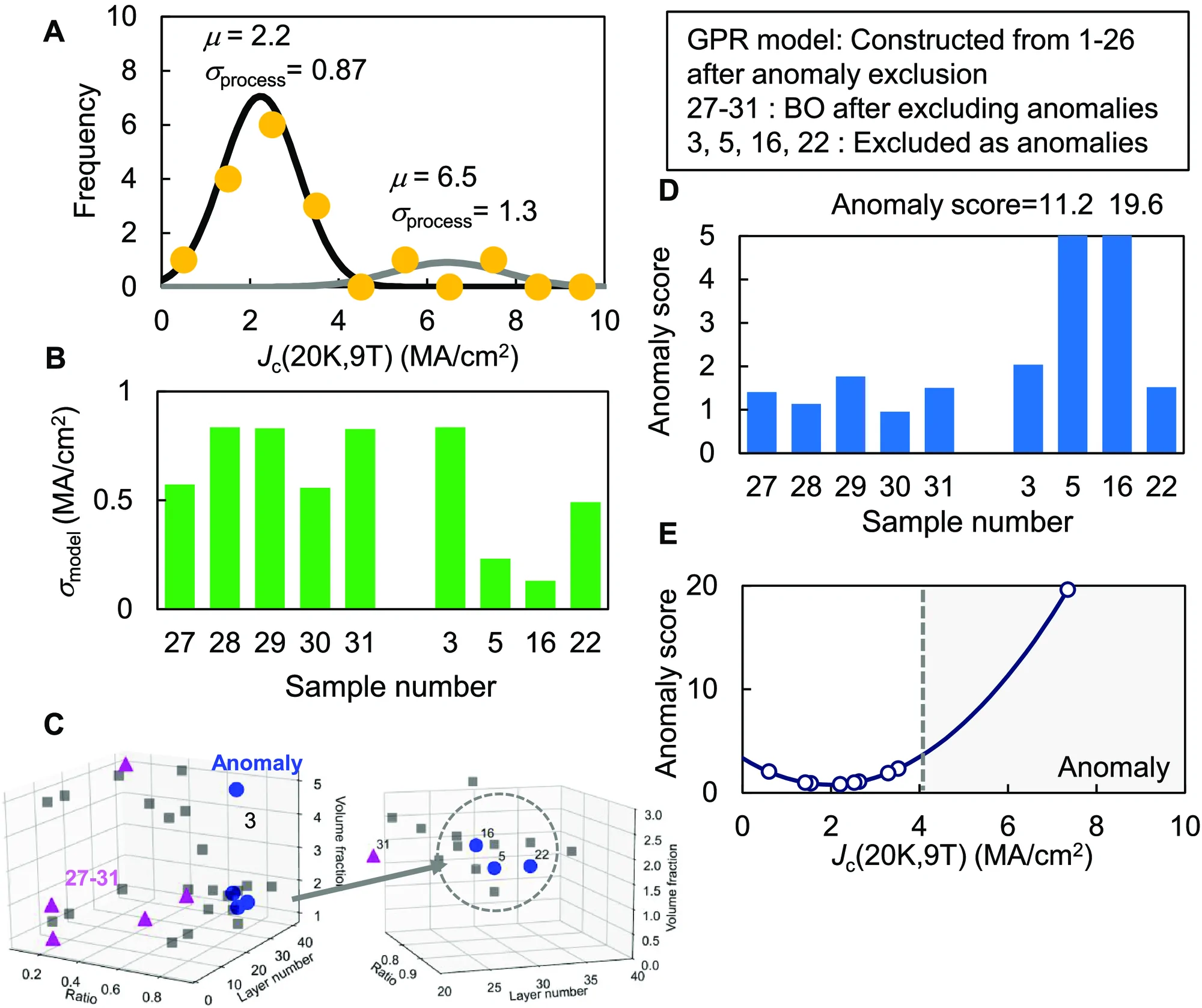
(A) Histogram of *J*_c_(20 K, 9 T), showing a bimodal distribution. (B) Standard deviation obtained from the GPR model (σ_model_) for each sample. (C) Distribution of data points with respect to the layer ratio, layer repetition number, and BHO volume fraction. The right panel shows an enlarged view of the left panel. (D) Anomaly score for each sample. (E) Anomaly score as a function of *J*_c_(20 K, 9 T). The anomaly score was calculated for samples prepared under the same conditions as Sample 16. Symbols represent the experimentally obtained *J*_c_ values and the corresponding anomaly scores. For B and D, σ_model_ and anomaly scores were evaluated for both the samples obtained by Bayesian optimization (BO) after anomaly exclusion (Samples 27–31) and the excluded anomalous samples (Samples 3, 5, 16, and 22).

Figure 3B shows σ_model_ for Samples 27–31 and 3, 5, 16, and 22, calculated using a model constructed from the other normal samples. σ_model_ is small for conditions 5, 16, and 22. Here, it should be noted that the *J*_c_^measured^ for Samples 5 and 16 is not used for constructing the model. To discuss the small σ_model_, Figure 3C shows the distribution of data points as functions of the layer ratio, layer repetition, and BHO volume fraction. A large number of data points are concentrated around conditions 5, 16, and 22. As the amount of data increases, the model accuracy improves through GPR, leading to a reduction in σ_model_.

To identify the anomalies based on data dispersion, a log-likelihood-based anomaly score is introduced. The anomaly score is determined based on the *J*_c_^measured^ and the two standard deviations (σ_process_ and σ_model_). σ_process_ corresponds to the dispersion of the *J*_c_ for fixed nominal conditions. The standard deviation (σ_model_) is evaluated in the GPR model to assess the reliability of the model. Figure 3D shows the anomaly scores. The log likelihood is used in Gaussian process to optimize hyperparameters and detect anomalies.^37–40^ In this study, we use the log likelihood to evaluate the anomaly in the process. Large anomaly scores were obtained for Samples 5 and 16, indicating that the measured *J*_c_ deviates from the predicted value. The anomaly score for Samples 3 and 22 is almost the same level as that for other samples, indicating that they may be treated as a weak fluctuation.

Since the nominal process experiment is the same, the σ_process_ is assumed to be constant regardless of conditions, and σ_process_ is 0.87 MA/cm^2^ for all the normal samples. It is not realistic to measure σ_process_ under all conditions. Since the process procedure is almost the same, using a common value of σ_process_ is reasonable. Figure S1 shows the anomaly scores for σ_process_ = 0.3, 0.87, and 1.2 MA/cm^2^. The anomaly scores do not vary significantly with σ_process_, confirming that σ_process_ does not affect which samples are identified as anomalies. The total standard deviation (σ_total_) includes the model uncertainty (σ_model_) and σ_process_. When the data amount is small, σ_model_ becomes large. In this case, the contribution of σ_process_ to σ_total_ is weak. Conversely, when the data amount is large, σ_model_ becomes small. In this case, σ_process_ can also be estimated more precisely because of the large amount of data.

Bayesian optimization consists of initialization, surrogate model construction, candidate selection based on acquisition functions, experimental evaluation, and an update of the model. For improved initialization, transfer-based Bayesian optimization has been proposed.^41^ For automated data acquisition and updating, robotic synthesis systems are promising. Numerous studies have focused on improving the surrogate modeling. The most basic surrogate model is the GPR. Methods incorporating failed experiments,^36^ missing data, multiple outputs,^42^ and subset optimization^43^ have also been developed. Thus, many studies focus on surrogate model design. On the other hand, the present scheme provides a candidate selection method that differs from conventional acquisition functions. We employ the log likelihood to recommend conditions worthy of further investigation. The scoring method based on the log likelihood is a well-known approach. In this study, a score based on the log likelihood is used to detect serendipitous events. Although anomalies are generally considered to be unfavorable, the scheme in this study is designed to identify favorable anomalies. This scheme enables the identification of unexpected and serendipitous events, whereas conventional acquisition functions are primarily designed to efficiently optimize the predicted performance under uncertainty. This study establishes a link between serendipitous events and the log likelihood and presents an overall framework for serendipity detection.

According to the bimodal distribution shown in Figure 3A, while a Gaussian distribution can account for the *J*_c_ distribution in the low-*J*_c_ samples, a different model is required for the high-*J*_c_ samples. Therefore, excluding samples with *J*_c_ > 4 MA/cm^2^ is the most effective approach, while the low-*J*_c_ samples with *J*_c_<4 MA/cm^2^ should be included in the GPR model. Figure 3E shows the anomaly score as a function of *J*_c_ for the Sample 16 condition. An anomaly score of 3.4, corresponding to 4 MA/cm^2^, can efficiently exclude the anomalously high-*J*_c_ samples. In this case, the excluded samples (Samples 5, 16) have anomaly scores of 11.2 and 19.6, whereas the other samples have an anomaly score of approximately 2. Since the anomaly scores differ significantly between the anomalous and normal samples, the threshold should be set within this gap.

In Figure 2C, Samples 3 and 22, as well as Samples 5 and 16, were excluded. Although this stricter exclusion reduces the amount of available data, the GPR model better represents the normal sample population because anomalous high-*J*_c_ samples are not included in the training data. Since Samples 5 and 16 are excluded, the exclusion method in Figure 2C is appropriate. Despite the reduction in the data quantity, stricter exclusion provides more reliable modeling. Thus, the Bayesian optimization shown in Figure 2C was performed by using a conservative exclusion strategy.

### Anomaly Detection Based on Physical Analysis

In this paragraph, we validate the data classification and anomaly identification through physical analysis. Figure 4A compares the experimentally measured *J*_c_ values (*J*_c_^measured^) with the average *J*_c_ estimated from the nanorod layer ratio (*r*) of YBCO + BHO (*J*_c_(SL)) to pure YBCO (*J*_c_(pure)): *J*_c_^avg^ = (1–*r*)*J*_c_(pure) + *rJ*_c_(SL). Here, SL denotes the YBCO + BHO single layer. The results are classified into four categories: *J*_c_^measured^/*J*_c_^avg^ < 0.5, 0.5 < *J*_c_^measured^/*J*_c_^avg^ < 1, *J*_c_^measured^/*J*_c_^avg^ = 1, and *J*_c_^measured^/*J*_c_^avg^ ≫ 1. The categories of *J*_c_^measured^/*J*_c_^avg^ = 1 and *J*_c_^measured^/*J*_c_^avg^ ≫ 1 include Samples 3, 22 and Samples 5, 16, respectively.

**Figure 4:**
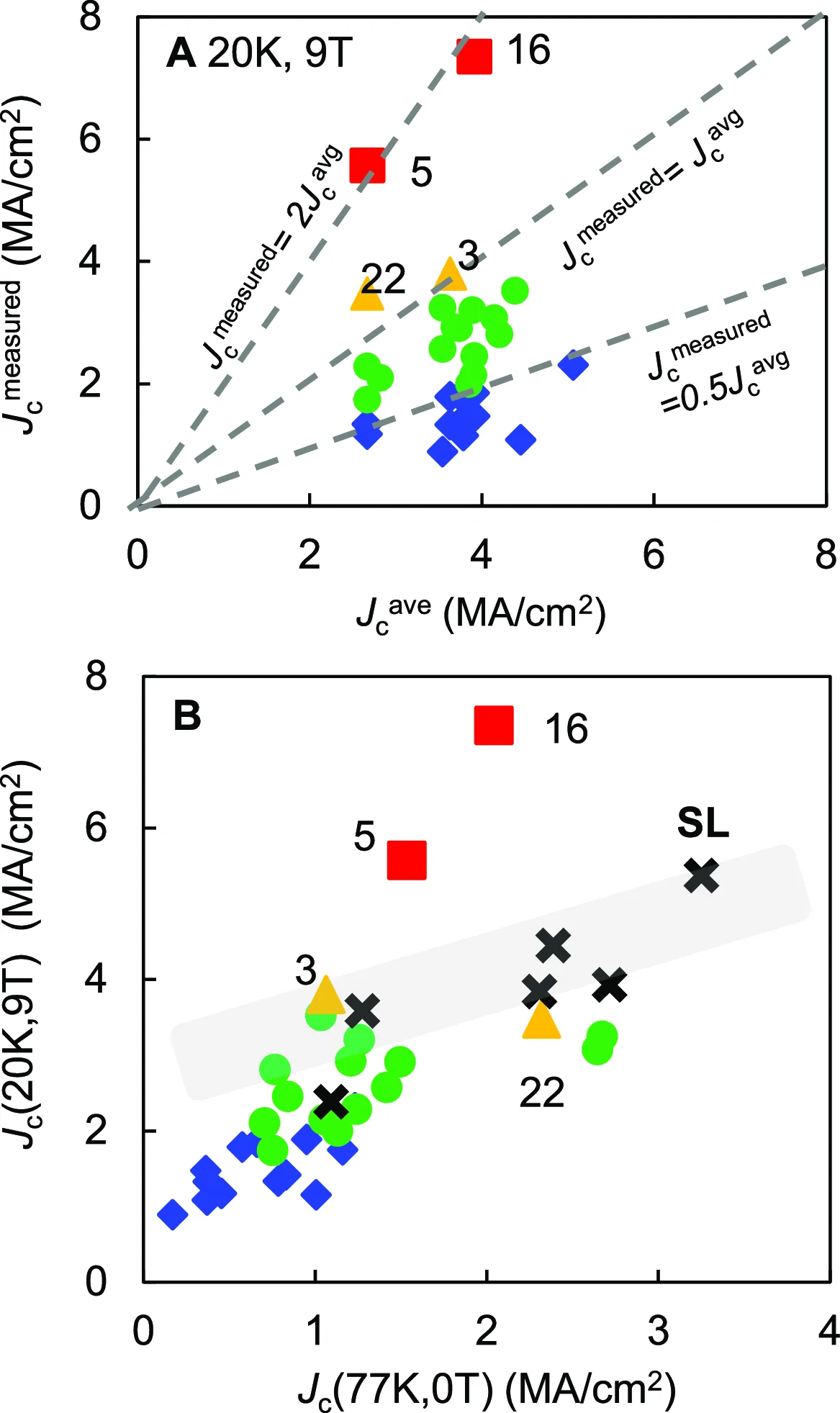
(A) Comparison of *J*_c_^measured^ and *J*_c_^avg^ at 20 K and 9 T. Dashed lines corresponding to *J*_c_^measured^/*J*_c_^avg^ = 0.5, 1, and 2 are shown. Four *J*_c_ categories are defined based on the ratio *J*_c_^measured^/*J*_c_^avg^. (B) Relationship between measured *J*_c_(20 K, 9 T) and measured *J*_c_ (77 K, 0 T). The results for the SL­(YBCO + BHO single layer) samples are also shown. The same symbols are used in (A, B).

To further analyze the *J*_c_ categories, *J*_c_(20 K,9 T) and *J*_c_(77 K,0 T) are compared in Figure 4B. This plot is sometimes referred to as the lift factor.^44,45^ The distribution of *J*_c_(20 K,9 T) and *J*_c_(77 K,0 T) depends on the multilayer structure. Samples 5 and 16, as well as Samples 3 and 22, fall into different categories in Figure 4B. For the same *J*_c_(77 K,0 T), the *J*_c_(20 K,9 T) is the highest in Samples 5 and 16, while in Samples 3 and 22 it is slightly higher than in the other films. Samples 3 and 22 show the same trend as that of the SL sample. The relationship between *J*_c_(20 K,9 T) and *J*_c_(77 K,0 T) is analyzed in terms of the *J*_c_^measured^/*J*_c_^avg^ values discussed in Figure 4A. The samples with *J*_c_^measured^/*J*_c_^avg^ < 1 exhibit low *J*_c_(20 K,9 T), whereas those with *J*_c_^measured^/*J*_c_^avg^ > 1 show high *J*_c_(20 K,9 T) at the same *J*_c_(77 K,0 T). The *J*_c_(20 K,9 T) at a given *J*_c_(77 K,0 T) increases with *J*_c_^measured^/*J*_c_^avg^.

For Samples 5 and 16, very large anomaly scores were observed in Figure 3D, and their *J*_c_ values deviate from the trend of the other samples in Figures 2C and 4A,B. For Samples 3 and 22, while the anomaly score was comparable to that of the normal samples as shown in Figure 3D, the *J*_c_ values for these samples fall within the higher end of the normal sample range in Figures 2C and 4A,B. Thus, the classification based on the anomaly score, the *J*_c_(20 K,9 T)-*J*_c_(77 K,0 T) relationship, and the ratio *J*_c_^measured^/*J*_c_^avg^ leads to a consistent conclusion.

### Occurrence Probability

Although Figure 3A was discussed to obtain the standard deviations, the occurrence probability of the two types of events, namely high *J*_c_ and low *J*_c_, is estimated. The Gaussian distribution is given by $\frac{1}{\sqrt{2\pi \sigma^{2}}}\exp\left(-\frac{{\left(x-\mu \right)}^{2}}{2\sigma^{2}}\right)$ . Figure 3A shows a bimodal distribution of *J*_c_, where μ_h_= 6.5 MA/cm^2^ and σ_h_ = 1.3 MA/cm^2^ for the high-*J*_c_ population (based on two data points), and μ_l_ = 2.2 MA/cm^2^ and σ_l_ = 0.87 MA/cm^2^ for the low-*J*_c_ population. The values for the high-*J*_c_ population should be regarded as indicative values because of the limited data set size. The probability density is expressed as

1 $$P=0.84\times \frac{1}{\sqrt{2\pi {\sigma_{l}}^{2}}}\exp\left(-\frac{{\left(J_{c}-\mu_{l}\right)}^{2}}{2{\sigma_{l}}^{2}}\right)+0.16\times \frac{1}{\sqrt{2\pi {\sigma_{h}}^{2}}}\exp\left(-\frac{{\left(J_{c}-\mu_{h}\right)}^{2}}{2{\sigma_{h}}^{2}}\right)$$

The low-*J*_c_ structure occurs approximately five times as frequently as the high-*J*_c_ structure. In the bimodal distributions, the ratio of the mean *J*_c_ value represents the pinning enhancement associated with the serendipitously discovered high-*J*_c_ structure. μ_h_/*μ*_l_ = 2.9 indicates that the high-*J*_c_ structure exhibits a 2.9-fold enhancement in *J*_c_. These values for occurrence probability (five times) and *J*_c_ ratio (2.9-fold) should be regarded as indicative estimates rather than statistically robust quantities, because they are based on a limited data set consisting of two samples in the high-*J*_c_ population.

*J*_c_ values significantly exceeding *J*_c_^avg^ were not observed in the nanocomposite multilayers.^27–29^ As will be discussed in the next section, spontaneous layer reconstruction along the thickness direction can occur and is key to the formation of the high *J*_c_ structure in this study. The layer unit is 7 nm in Sample 16. In contrast, in the previous multilayers, the layer thickness was sufficiently large (typically, layer unit = [YBCO spacer] + [YBCO + BHO] > 40 nm),^27–29^ which suppresses spontaneous layer reconstruction through diffusion along the thickness direction. In addition, higher volume fractions of nanorods were introduced in previous studies, affecting the nanorod correlation across the spacer layer.

### Multilayer Periodicity and Nanorod Correlation

Although anomaly detection is possible based on the *J*_c_ values through data science or physical analyses, clarifying the nanostructure and pinning mechanisms underlying these anomalies will help to verify the anomaly identification and optimize the film fabrication process. Here, three samples were compared. Sample 16 exhibits a high *J*_c_, whereas Sample 16–2 corresponds to the low-*J*_c_ structure in the bimodal distribution shown in Figure 3A. In addition, the YBCO + BHO (2 vol %) single layer (SL) is included for comparison.

Figure 5 shows cross-sectional scanning transmission electron microscopy (STEM) images. The SL sample exhibits nanorods extending throughout the film, with a diameter and spacing of 6 and 25 nm, respectively. Sample 16 exhibits a well-defined multilayer structure. The cross-sectional image of anomalous Sample 16 exhibits two characteristic features: multilayer periodicity and nanorod correlation. Sample 16 consists of layers with thicknesses of 14.3 ± 2.6 nm for the YBCO+BHO layer and 4.5 ± 1.1 nm for the pure YBCO layer, both of which are thicker than the designed values (5.7 and 1.4 nm, respectively). According to Figure S2 and Table S3, the number of repetitions is 11, which is smaller than the designed value of 28. In contrast, Sample 16–2 does not exhibit a well-defined multilayer structure, where the BHO nanorods are distributed randomly. The nanostructures of Samples 16 and 16–2 differ despite having the same volume fraction and designed layer thickness.

**Figure 5:**
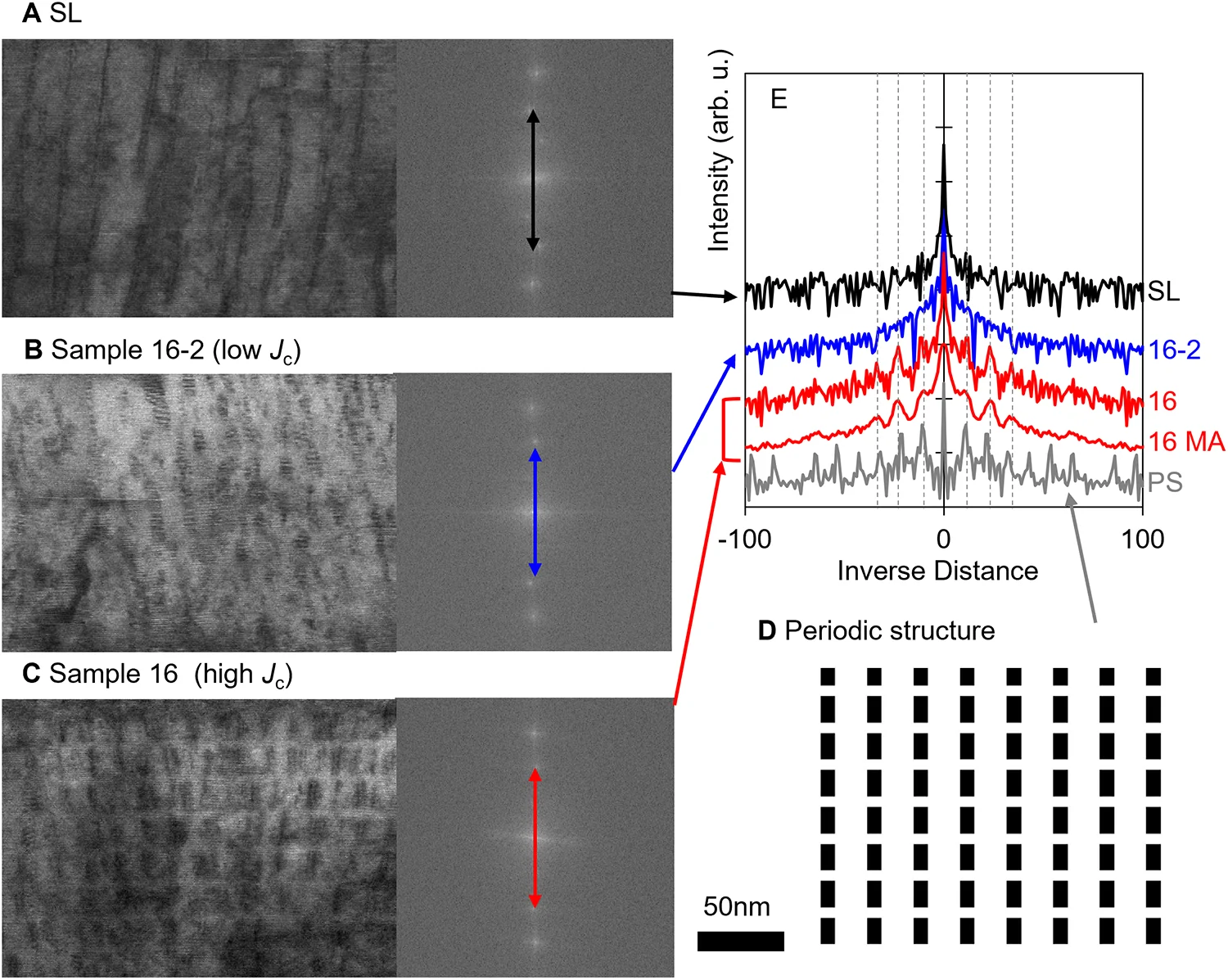
(A–C) Cross-sectional STEM images. Cross-sectional STEM images of (A) the SL, (B) Sample 16, and (C) Sample 16–2, respectively. (D) Periodic structure with the same layer thickness and contrast size. (E) Intensity profiles of the Fourier-transformed spectra for the SL (black), Sample 16–2 (blue), Sample 16 (red), and the periodic structure (gray). The moving-average (MA) of the intensity for Sample 16 is indicated by 16 MA, and the periodic structure is represented by PS.

The YBCO/BHO heterointerface is energetically unfavorable. To reduce the total system energy, the interfacial area between YBCO and BHO was reduced. However, kinetic limitations and restricted diffusion suppress the transformation toward a more stable structure. When the layer thickness is smaller than the diffusion length, diffusion occurs across the layers along the thickness direction, resulting in disruption of the multilayer structure. In contrast, when the diffusion length is shorter than the layer thickness, the system cannot kinetically evolve toward a more stable structure with reduced interfacial area. The YBCO spacer layers in the designed multilayer structure are only one or two unit cells thick in Sample 16, allowing diffusion across the layers and promoting spontaneous structural reorganization toward a more thermodynamically stable state. This type of self-organization is similar to that observed in multilayer films prepared using multiple targets.^46^

The vertical correlation of the nanorods is another key factor in understanding the multilayer structure and its pinning properties. To analyze the nanorod alignment along the thickness direction, Fourier transforms of the cross-sectional images are shown in Figure 5. For the SL and Sample 16–2, the intensity monotonically decreases with increasing inverse distance. In contrast, Sample 16 exhibits periodic peaks, which are clearer in moving-averaged curves. To confirm the periodicity, the Fourier transform of artificially generated ordered boxes with the same size and spacing is also shown. The peak positions are nearly identical to those of Sample 16, indicating that the peaks originate from the periodicity of the contrast. The peak shapes and heights differ from those of the ideal periodic structure, and higher-order peaks are obscured. These results suggest an imperfect periodicity and a weakly correlated nanorod structure in Sample 16. The mechanism of the vertical alignment has been attributed to strain, which has been previously discussed for the vertical alignment of nanorods and quantum dots in thin films.^27,47,48^

Figure 6A–C shows plan-view STEM images of the SL, Sample 16, and Sample 16–2, respectively, to analyze the nanorod correlations. All films exhibit contrast indicating the presence of nanorods. The nanorods have a diameter of 5–10 nm and a spacing of 20–40 nm. The detailed distribution of nanorods differs between the films. The SL sample exhibits slightly elongated contrasts, attributed to the tilting of nanorods observed in the cross-sectional images. In sample 16–2, the circular contrasts are finely dispersed. In contrast, Sample 16 exhibits a characteristic nanostructure. Magnified images, shown in Figure 6D,E, reveal two nanorods located in close proximity and partially overlapping, indicating weak correlation across YBCO spacer layers. This structure is not observed in the SL and Sample 16–2, even in the magnified images shown in Figure S3.

**Figure 6:**
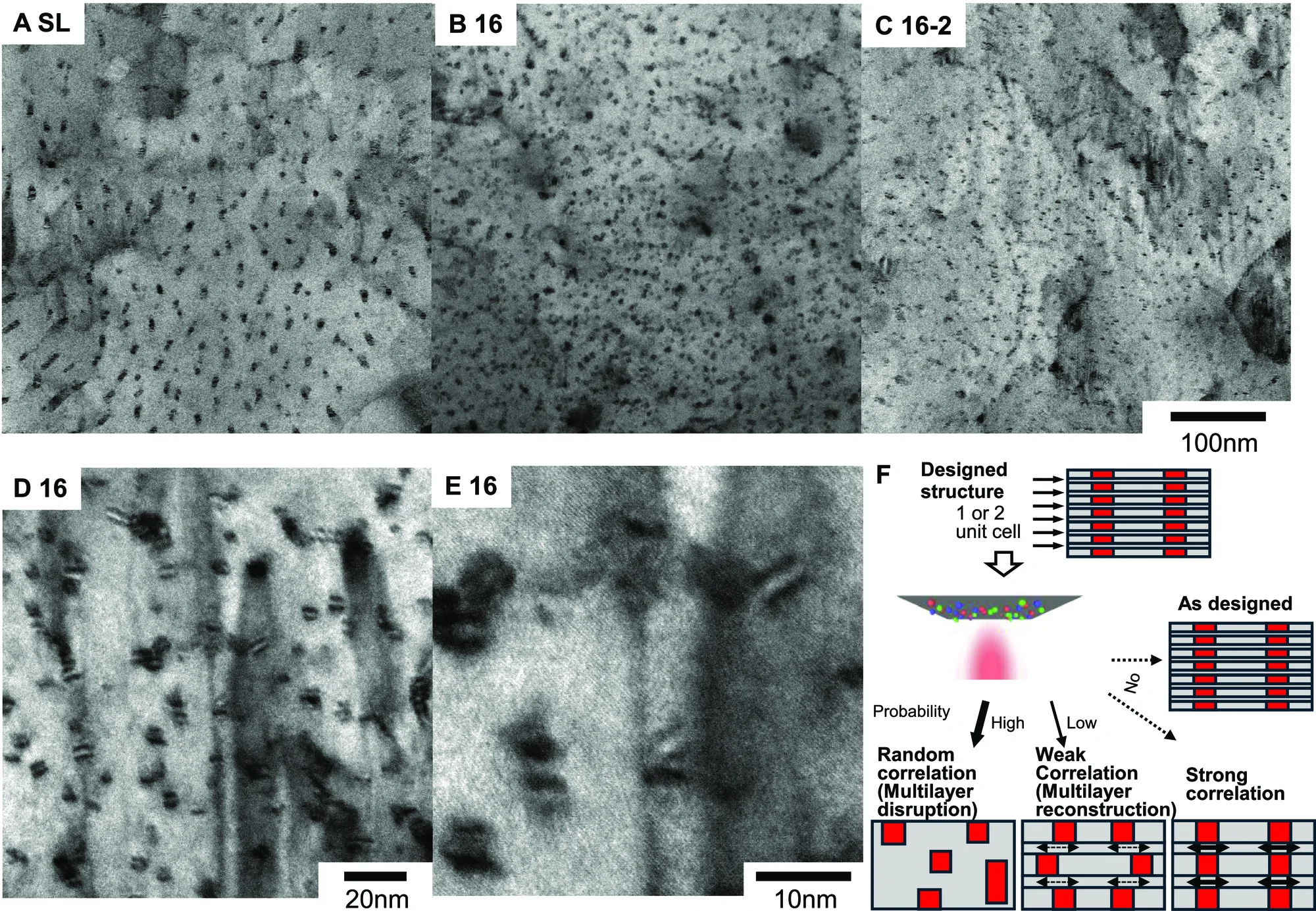
(A–C) Plan-view STEM images of (A) the SL (YBCO+BHO single layer), (B) Sample 16 , and (C) Sample 16-2, respectively. (D, E) Magnified images of Sample 16. Sample 16 exhibits weakly correlated nanorods. (F) Schematic illustration of nanorod correlation and misalignment. Weakly correlated or random nanorods are formed, whereas the designed structure does not form.

Figure S4 shows a cross-sectional STEM image of sample 5, which belongs to the high-*J*_c_ population in Figure 3A. The STEM image confirms the presence of weakly correlated nanorods. In addition, because the layer repetition number was 10, spontaneous multilayer reconstruction occurred. The FFT also shows satellite peaks. These features are similar to those of Sample 16. However, the periodicity of the reconstructed layers is slightly weaker than that in Sample 16, and consequently, higher-order satellite peaks are less distinct. Figure S5 shows a plan-view STEM image of Sample 5. Similar to Sample 16, two nanorods in Sample 5 are located in close proximity and partially overlap. Thus, the results obtained for Sample 5 are consistent with the weakly correlated nanorod structure and enhanced *J*_c_ behavior (as discussed in the next section) observed in Sample 16. The conclusions drawn from Sample 16 were also confirmed in a second independently deposited sample (Sample 5), supporting the present conclusion beyond a single-sample observation.

The schematic illustration in Figure 6F summarizes the nanorod correlations in multilayer structures. The designed structure contains one or two unit-cell-thick YBCO spacer layers. Due to the instability of this design, spontaneous layer reconstruction occurred over several layer units, resulting in weak and random correlations between nanorods. These correspond to Samples 16 and 16-2, respectively, as determined from the STEM observations. In Sample 16, when the well-defined multilayer is spontaneously formed, the growth of nanorods is weakly influenced by those in the underlying layer, resulting in weakly correlated nanorods.

### *J*_c_ Characteristics

To discuss the differences in vortex pinning among the *J*_c_ categories, Figure 7A–C compares the *J*_c_-*B* characteristics for the SL sample, the anomalous samples with *J*_c_^measured^ ≫ *J*_c_^avg^ (Samples 5, 16), and the normal sample with *J*_c_^measured^ < *J*_c_^avg^ (=3.9 MA/cm^2^) (Sample 16-2). Samples 5 and 16, which occur with a low probability, exhibit similar *J*_c_-*B* characteristics. An anomalously high *J*_c_ is observed in the independently deposited samples.

**Figure 7:**
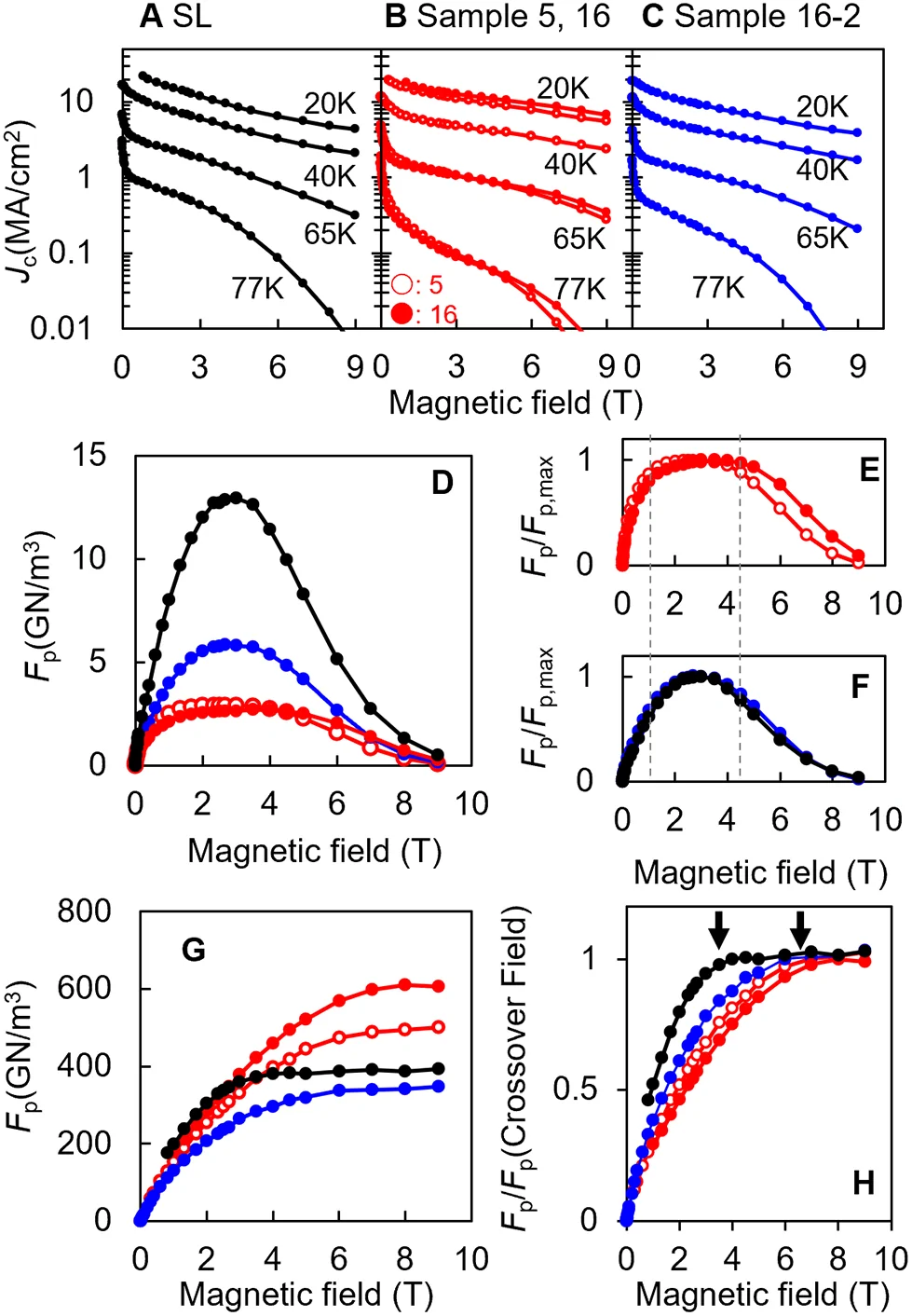
*J*_c_ and pinning force density (*F*_p_) characteristics of the films. Magnetic field dependence of *J*_c_ at 77, 65, 40, and 20 K for (A) SL, (B) samples 5 and 16, and (C) sample 16-2. Magnetic field dependence of *F*_p_ at (D) 77 K and (G) 20 K. Normalized *F*_p_ at 77 K (E, F) and 20 K (H). Arrows in (H) indicate where *F*_p_ becomes constant. The same symbols and colors are used throughout panels (A–H): black solid symbols (SL), red open symbols (Sample 5), red solid symbols (Sample 16), and blue solid symbols (Sample 16-2).

Figure 7D–F shows the pinning force density (*F*_p_ = *J*_c_ × *B*) and normalized *F*_p_ as a function of magnetic field at 77 K. *F*_p_ was normalized to the maximum *F*_p_ in Figure 7E,F. A peak in *F*_p_ is observed at 3 T for the SL and sample 16-2, whereas samples 5 and 16 exhibit a broad *F*_p_ peak spanning 2–5 T. It should be noted that the broad peak in Samples 5 and 16 extends to higher magnetic fields than the *F*_p_ peak of the SL. The matching field is a very important parameter in the nanorod pinning. The matching field (*B*_Φ_) is defined as the magnetic field at which all nanorods are occupied by vortices, and is given by *nϕ*_0_, where *n* is the nanorod density and ϕ_0_ is the flux quantum. The matching-field effect depends not only on the nanorod spacing but also on the dispersion of nanorod spacing.^49^ However, the broad matching-field effect observed in Sample 16 is absent in the *F*_p_–*B* curve of the SL, suggesting that nanorod pinning cannot explain the behavior of Sample 16.

Figure 7G,H shows the *F*_p_ and normalized *F*_p_ as a function of magnetic field at 20 K. *F*_p_ increases with magnetic field up to the crossover field and then reaches a nearly constant value regardless of sample. Here, the crossover field is defined as the magnetic field at which *F*_p_ becomes constant as the magnetic field increases. *F*_p_ was normalized to the *F*_p_ at the crossover field. The crossover field was defined as the magnetic field where $\left|\frac{dF_{p}}{dB}\right|<0.05{\left|\frac{dF_{p}}{dB}\right|}_{B\approx 0T}$ , indicating that *F*_p_ becomes nearly independent of magnetic field. For the SL, the crossover field corresponds to the magnetic field where the *F*_p_ peak was observed at 77 K, indicating that both the *F*_p_ peak field and the crossover field correspond to the matching field. The crossover field for Sample 16-2 is slightly higher than the *F*_p_ peak field at 77 K, suggesting that its pinning mechanism differs from that of the SL. In Sample 16, the crossover field is higher than that of Sample 16-2 and lies outside the range of the broad *F*_p_ peak at 77 K. These observations in the *F*_p_-*B* curves suggest that the vortex behavior in Sample 16 differs from that of conventional nanorod systems, namely the SL in this study.

### Pseudomatching of Vortices

Pinning mechanisms are often interpreted in a suggestive manner based on experimental observations. Such analyses are typically based on morphological factors, such as spacing, elongation, and size as in matching field effect analysis. The broad matching-field effect and the anomalous *J*_c_(20 K, 9 T)–*J*_c_(77 K, 0 T) relationship cannot be explained by variations in nanorod density. To fully understand these features, not only the nanorod structure but also the vortex configuration should be considered. Vortex energies were calculated in this study to analyze vortex configurations. The system energy of vortices is given by

2 $$E=\int_{V}U_{p}+\varepsilon_{l}+\sum_{i\neq j}\frac{{\phi_{0}}^{2}}{4\pi \mu_{0}\lambda^{2}}K_{0}\left(\frac{r_{i,j}}{\lambda }\right)dr$$

The first, second, and third terms correspond to the pinning potential, line tension, and vortex–vortex interactions, respectively. For *B* > *B*_Φ_, three possible situations can arise depending on the nanorod configuration, as shown in Figure 8A. The stability of vortex configurations is evaluated by comparing the energies relative to configuration A, as shown in Figure 8.

**Figure 8:**
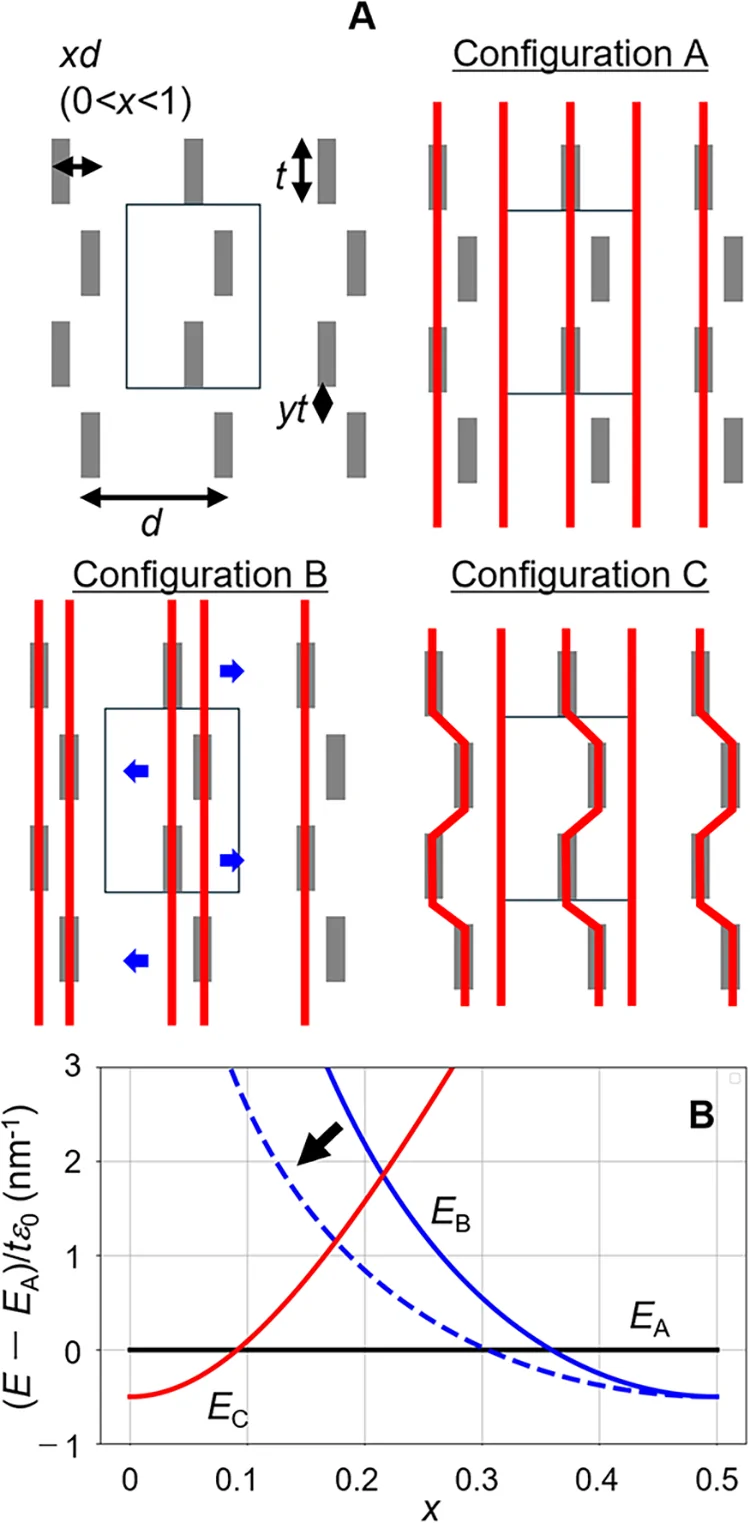
(A) Definition of the nanorod structure and the periodic unit. The energy was calculated for the periodic unit indicated by the solid square. Vortex configurations (A–C) correspond to the regular array, pseudomatching, and staircase arrangements, respectively. (B). System energy as a function of the nanorod misalignment *x*. The dashed curve represents the result obtained using half the elastic contribution, schematically corresponding to a relaxed state. The energy was normalized by *t*ε_0_. *x* is a dimensionless quantity representing the distance normalized by *d*.

For *x* < 0.1, the staircase vortex configuration (configuration C) is stable. In contrast, for *x* > 0.35, all vortices are pinned by short nanorods with an energy increase due to repulsive vortex–vortex interaction (configuration B). As shown in Figure 6E, the nanorod misalignment is approximately 10 nm, corresponding to *x* ≈ 0.4. More stable configuration may be possible for the pseudomatching configuration. The nanorod misalignment occurs randomly around the underlayer nanorods, allowing the repulsive force acting on the unpinned vortex segments to relax. As illustrated by the arrows in configuration B, the repulsive force on the unpinned vortices can relax as they move farther away from the pinned vortices. These effects reduce the overall system energy as shown by a dashed curve in Figure 8. Figure S6 shows the parameter dependence (α, *t*, *d*, *y*) of the energy and vortex configurations. Independent of the parameters, vortex configurations C, A, and B appear with increasing *x*. Thus, vortex configuration B appears regardless of the parameters and structural variations. This indicates that a pseudomatching configuration (configuration B) is realized in the YBCO+BHO/YBCO multilayers. This reinforces and supports the present conclusion on pseudomatching field effect.

In the conventional matching-field effect, which is determined by the structure, each nanorod accommodates one vortex. On the other hand, the competition among pinning energy, vortex–vortex interactions, and vortex line tension enables vortices to be more effectively accommodated by the nanorods in each layer. Due to nanorod misalignment across the layers, a larger number of vortices than the nominal accommodation capacity of the SL become directly pinned by nanorods, resulting in an increased effective matching field. This phenomenon corresponds to a pseudo-high-density nanorod structure that induces a pseudomatching effect. Thus, pseudomatching arises not only from the pinning structure itself but also from the vortex configuration. The competition among pinning, vortex interactions, and line tension induces various vortex behaviors.^50–54^ Therefore, introducing nanorod misalignment is a promising strategy for achieving enhanced pinning mechanisms.

### Application of the Present Scheme and Pinning Structure to Coated Conductors

Although the energy-based analysis can explain the experimental observations, it remains suggestive, rather than fully predictive. Under the current limitations of vortex modeling, the combination of experimental exploration and data-driven approaches is effective for pinning design, and serendipity engineering enables the discovery of unexpected improvements. Therefore, the combination of experimental exploration guided by data-driven methods and subsequent analysis to gain insights into the underlying mechanisms, as demonstrated in this study, represents a promising framework for studying such problems.

This study focuses on low-probability phenomena. Sample 5 exhibits similar properties to Sample 16 in Figure 7, and a structure similar to that of Sample 16 was observed in Sample 5, as shown in Figures S4 and S5. However, its occurrence probability is low, as shown in Figure 3A. This study demonstrates a scheme to avoid overlooking low-probability serendipitous events. The discovery of the nanostructure and its pinning mechanism, together with the proposed approach, constitutes one of the main conclusions of this study. The discovered structure is promising for improving the *J*_c_. To increase the occurrence probability for industrial applications, further optimization studies are required. Control of diffusion and driving force based on nanorod materials and growth rates is required to promote spontaneous layer reconstruction with a high occurrence probability.

## Conclusion

The serendipitous discovery of nanostructures and pinning mechanisms in YBCO nanocomposite multilayers was achieved using a data-driven serendipity detection framework that combines Bayesian optimization and anomaly detection. The YBCO + BHO/YBCO nanocomposite multilayer films were fabricated using pulsed laser deposition. Anomalously high *J*_c_ values were detected by using an anomaly score defined by the log likelihood. Because of the instability of YBCO layers one or two unit cells thick, the designed nanostructure did not form in the anomalously high-*J*_c_ films, and spontaneous layer reconstruction occurred across several layer units. In these films, the weakly correlated nanorods induce the pseudomatching effect of vortices, resulting in a 2.9-fold enhancement in *J*_c_, as estimated from the comparison of the means of the bimodal distributions. This reveals an unexpected optimum in pinning energy, elastic interactions, and line tension that is not found in conventional nanorods or nanoparticles. In addition to the vortex pinning aspect, this outcome represents an unexpected improvement on the original experimental design and benefits from serendipity engineering. The present data-driven approach based on anomaly detection is highly effective in enabling serendipitous discoveries of nanostructures and physical mechanisms in nanoscience and nanoengineering fields.

## Methods

### Sample Fabrication and Measurement of Superconducting Property

YBCO films were fabricated using reel-to-reel pulsed laser deposition (PLD). The substrates were Ion-Beam-Assisted Deposition (IBAD) templates with a CeO_2_ top layer. The deposition temperature, oxygen pressure, and laser pulse frequency were set to 870 °C, 53 Pa, and 10 Hz, respectively. Pure YBCO and YBCO + BHO targets were alternately ablated to form multilayers of designed thickness. The volume fraction of BHO, layer ratio, and number of layer repetitions shown in Figure S7 were varied while keeping the total designed thickness constant at 200 nm. The total film thickness was confirmed to be approximately 200 nm by scanning transmission electron microscopy (STEM) for selected samples.

After confirming the film orientation via X-ray diffraction, silver electrodes were sputtered, and laser cutting was performed to prepare a 1 mm long, 50 μm wide bridge. Resistance–temperature and current–voltage measurements were carried out using Physical Property Measurement System (PPMS) to determine critical temperature (*T*_c_) and critical current density (*J*_c_), with a criterion of 1 μV/cm. *J*_c_ was measured at 77, 65, 40, and 20 K under magnetic fields ranging from 0 to 9 T.

### Structure Characterization and Analysis

STEM was used to observe the cross-section and plan-view nanostructures of the films. Fourier transformation was performed on the cross-sectional STEM images, which had a resolution of 509 × 359 pixels. A 2D fast Fourier transform (FFT) was applied using ImageJ software. The power spectrum along the vertical axis was analyzed to evaluate the *c*-axis alignment.

### Bayesian Optimization

The subsequent multilayer fabrication conditions were proposed using Bayesian optimization. The corresponding data sets are shown in Table S1. Bayesian optimization was performed using PHYSBO, a GPR and Bayesian optimization library in Python.^55^ A Gaussian kernel function was used, and the hyperparameters were automatically optimized by the library. The volume fraction of BHO, layer ratio, and layer repetition was used as an explanatory parameter. The first 12 samples (Samples 1–12) were used as the initial data set for the GPR model. Conventional Bayesian optimization was then performed for Samples 13–26. The initial data set was obtained for various BHO volume fractions (1.5 vol %, 5 vol %), layer ratios, and layer thicknesses, corresponding to sample numbers *n* = 1–12. After the GPR model was constructed from the initial data, Bayesian optimization was performed using two acquisition functions: expected improvement (EI) and Thompson sampling (TS). Candidate selection was performed using PHYSBO with the default acquisition settings.^55^ Since PPMS can measure two samples simultaneously and the fabrication and measurement of YBCO films require significant time, two samples recommended by EI and TS were evaluated in parallel. When experimental failure due to wiring was observed, the model was updated with one additional data point. The results from these experiments were subsequently used to update the GPR model. Samples *n* = 13–26 were obtained by conventional Bayesian optimization without excluding anomalies.

### Bayesian Optimization Excluding Anomalies

Some anomalous *J*_c_ values are included in the results. Therefore, it is necessary to exclude anomalies from the GPR model to improve prediction accuracy. We developed an improved GPR–Bayesian optimization framework that detects and excludes anomalous data. After data points with large anomaly scores were excluded based on the initial GPR model, Bayesian optimization was performed. If *y_n_* is regarded as normal, the next candidate *x*_n+1_ is recommended through Bayesian optimization for {*y*_1_, *y*_2_,..., *y*_*n*‑1_, *y_n_*}. Subsequently, the GPR model is constructed using {*y*_1_, *y*_2_,..., *y*_*n*‑1_, *y_n_*, *y*_*n*+1_} and the next candidate *x*_*n*+2_ is again recommended through Bayesian optimization. This process corresponds to the conventional Bayesian optimization.

Conversely, if *y_n_* is regarded as an anomaly, a temporary GPR model is constructed using {*y*_1_, *y*_2_,..., *y*_*n*‑1_, *y_n_*}, and the next candidate *x*_n+1_ is recommended. In the subsequent step (*n* + 2), the GPR model is reconstructed using {*y*_1_, *y*_2_,..., *y*_*n*‑1_, *y*_*n*+1_}, and the next candidate *x*_*n*+2_ is then recommended through Bayesian optimization. When an anomaly is detected, the anomaly is temporarily included in the data set to update the model but is later excluded to prevent bias accumulation in subsequent iterations.

After excluding anomalies from the initial data set (Samples 1–26), this procedure was applied to samples 27–31. Although anomaly identification should ideally be based on anomaly scores, a tentative exclusion criterion was adopted in this case and its validity was subsequently confirmed.

### Anomaly Analysis

To detect anomalies, the anomaly score was defined as follows:

1. Determine the standard deviation originating from process variation, σ_process_.
2. Determine the predicted mean value (μ) from the GPR model. The standard deviation originating from the GPR model, σ_model_, is also obtained to evaluate the reliability of the predicted mean value.
3. Overall standard deviation is given by $\sigma =\sqrt{{\sigma_{\mathrm{model}}}^{2}+{\sigma_{\mathrm{process}}}^{2}}$ .
4. Calculate the anomaly score (A) as $A=-\mathrm{Ln}\left(P\right)=-\mathrm{Ln}\left(\frac{1}{\sqrt{2\pi \sigma^{2}}}e^{-{\left(x-\mu \right)}^{2}/2\sigma^{2}}\right)$ .

### Vortex Energy Calculation

To discuss the vortex configuration at high magnetic fields, magnetic fields exceeding the matching field are considered. Figure 8 illustrates the corresponding vortex configurations:

*Configuration A: Regular Array* Some vortices are pinned by the nanorods, while others are elastically fixed without direct pinning. In this case, because not all pinning sites are occupied, the pin potential is not fully utilized.

*Configuration B: Pseudomatching* Vortices are pinned by the nanorods in a configuration that maximizes the number of directly pinned vortices. In this case, the excess repulsive energy arising from vortex–vortex interactions is increased.

*Configuration C: Staircase* vortices are pinned by the nanorods in a staircase arrangement to maximize the pinned volume.^56,57^ Other vortices are stabilized through vortex interactions. In this case, the line energy increases because the vortex length becomes longer. Here, we assume that the line energy is the dominant contribution to the energy increase.

We compare the energies of the three configurations. The energy is calculated for a solid square region representing a periodic unit in Figure 8. According to eq ([Disp-formula eq2]), the system energies for configurations A, B, and C can be expressed as follows

3 $$E_{A}\left(x\right)=tU_{p}+\left(4t+4\mathrm{yt}\right)\times \frac{{\phi_{0}}^{2}}{4\pi \mu_{0}\lambda^{2}}\times {2K}_{0}\left(\frac{d}{2\lambda }\right)+\left(4t+4\mathrm{yt}\right)\varepsilon_{l}$$

4 $$E_{B}\left(x\right)=2tU_{p}+\left(4t+4\mathrm{yt}\right)\times \frac{{\phi_{0}}^{2}}{4\pi \mu_{0}\lambda^{2}}\times \left(K_{0}\left(\frac{\mathrm{xd}}{\lambda }\right)+K_{0}\left(\frac{d-\mathrm{xd}}{\lambda }\right)\right)+\left(4t+4\mathrm{yt}\right)\varepsilon_{l}$$

5 $$E_{c}\left(x\right)=2tU_{p}+\left(4t+4\mathrm{yt}\right)\times \frac{{\phi_{0}}^{2}}{4\pi \mu_{0}\lambda^{2}}\times {2K}_{0}\left(\frac{d}{2\lambda }\right)+\left(4t+2\sqrt{x^{2}d^{2}+y^{2}t^{2}}+2\mathrm{yt}\right)\varepsilon_{l}$$

Here, *t* and *d* are the length and spacing of the nanorods, respectively; *xd* and *yt* are the nanorod misalignment and the spacer layer thickness, respectively; μ_0_ and ϕ_0_ are the permeability and flux quantum, respectively; λ and ξ are the penetration depth (∼200 nm in YBCO) and coherence length (2 nm in YBCO); and $\varepsilon_{0}=\frac{{\phi_{0}}^{2}}{4\pi \mu_{0}\lambda^{2}}$ . The pin potential, *U*_*p*_ *= α*ε_0_, where α is the pinning efficiency. *K*_0_(*x*) is the modified Bessel function of the second kind. The calculation of the vortex–vortex interaction term, expressed by the modified Bessel function, was performed considering only the first nearest-neighbor vortices. The line tension energy is expressed as ε_l_ = ε_0_ ln­(λ/ξ). Since unpinned vortices can relax in Configuration C, the vortex interaction term in *E*_c_(*x*) was estimated using the minimum-interaction-energy vortex configuration.

To discuss the stable configuration, the energy relative to *E*_A_(x) is analyzed. By normalizing with *t*ε_0_, the following equations are obtained

6 $$\frac{E_{B}\left(x\right)-E_{A}\left(x\right)}{t\varepsilon_{0}}=\alpha +4\left(1+y\right)\times \left(K_{0}\left(\frac{\mathrm{xd}}{\lambda }\right)+K_{0}\left(\frac{d-\mathrm{xd}}{\lambda }\right)-{2K}_{0}\left(\frac{d}{2\lambda }\right)\right)$$

7 $$\frac{E_{C}\left(x\right)-E_{A\left(x\right)}}{t\varepsilon_{0}}=\alpha +2\left(\sqrt{\frac{d^{2}}{t^{2}}x^{2}+y^{2}}-y\right)\ln\left(\frac{\lambda }{\xi }\right)$$

The energies are discussed relative to *E*_A_(*x*), the energy of configuration A. Figure 8 shows the system energy as a function of *x*, which represents the nanorod misalignment. Pinning efficiency­(α), nanorod length (*t*), spacer layer thickness (*yt*), and nanorod spacing (*d*) are variable parameters (Figure S6). The energy calculations in Figure 8 were performed for the following parameters: α = 0.5, *y* = 0.5 ((spacer layer thickness) = 1/2 × (nanorod layer thickness)), *d* = 25 nm (*d*/λ = 1/8), λ/ξ∼100, and *t* = 10 nm (*d*/*t* = 2.5). *d* = 25 nm corresponds to a matching field of 3.3 T, where an *F*_p_ peak was observed in the SL.

## Supplementary Material



## Notes

Data set for Bayesian optimization, data set of the samples shown in Figure 3A, structural parameters of multilayers of sample 16, anomaly score for each sample for different σ_process_, cross-sectional STEM images of sample 16, plan-view STEM images of the SL and sample 16–2, cross-sectional STEM image for sample 5, plan-view STEM image for sample 5, system energy as a function of nanorod alignment for various parameters, and schematic of the designed layer structure (PDF)
