# Supplementary material for: Data-Driven Serendipitous Discovery of a Self-Organized Nanostructure for Enhanced Pinning in Superconducting Films
Source: ACS Nano. 2026 Aug 3;20(32):22635–47. doi: 10.1021/acsnano.6c05455 (PMC13492474; doi:10.1021/acsnano.6c05455)
Supplement: Supplementary file 1 [file nn6c05455_si_001.pdf]

Supporting Information

Data-Driven Serendipitous Discovery of a Self-Organized Nanostructure for Enhanced Pinning in Superconducting Films

Tomoya Horide[1,*], Shunta Ito[1], Ataru Ichinose[2], Yutaka Yoshida[1]

[1] Department of Electrical Engineering, Nagoya University, Furo-cho, Chikusa, Nagoya 464-8603, Japan

[2] The Grid Inovation Research Laboratory, Central Research Institute of Electric Power Industry, Yokosuka, Kanagawa 240-0196, Japan

* horide @nuee.nagoya-u.ac.jp

Table 1 Dataset for Bayesian optimization. Some coupled data are missing due to experimental failure. EI and TS denote Expected Improvement and Thompson Sampling acquisition functions, respectively.

Number	Ratio	Layer repetition	Volume fraction	YBCO+ BHO thickness (nm)	Pure thickness (nm)	J_c (77K, 0T) (MA/cm^2)	J_c (20K, 9T) (MA/cm^2)	T_c (K)	Acquisition function
1	0.8	10	5	16	4	0.6	1.8	87.0	(Initial)
2	0.2	10	5	4	16	1.2	1.7	89.4	(Initial)
3	0.8	30	5	5	1	1.1	3.8	88.2	(Initial)
4	0.2	10	1.5	4	16	1.4	2.6	89.1	(Initial)
5	0.8	30	1.5	5	1	1.5	5.6	87.0	(Initial)
6	0.2	30	1.5	1	5	0.2	0.9	86.6	(Initial)
7	0.8	4	5	40	10	0.4	1.3	88.6	(Initial)
8	0.8	4	1.5	40	10	0.8	1.7	88.9	(Initial)
9	0.8	10	1.5	16	4	0.8	1.3	89.6	(Initial)
10	0.2	4	5	10	40	1.0	1.2	90.2	(Initial)
11	0.2	30	5	1	5	0.8	1.4	89.2	(Initial)
12	0.2	4	1.5	10	40	2.7	3.2	90.3	(Initial)
13	0.8	34	2	5	1	1.2	2.3	89.7	EI
14	0.7	26	1.5	5	2	0.7	2.1	88.6	TS
15	0.8	30	1	5	1	1.2	2.3	89.2	EI
16	0.8	28	2	6	1	2.0	7.4	87.6	EI
17	0.8	26	2	6	2	0.8	2.5	88.1	EI
18	0.6	12	5	10	7	1.2	2.9	89.5	TS
19	0.8	30	2	5	1	0.7	1.8	89.3	EI, TS
20	0.8	28	1.5	6	1	0.5	1.2	89.3	EI
21	0.9	36	2	5	1	0.4	1.5	86.9	TS
22	0.8	34	1.5	5	1	2.3	3.5	89.8	EI
23	0.6	32	3	4	3	0.8	2.8	87.6	TS
24	0.7	28	2	5	2	1.1	2.1	89.7	EI
25	0.6	26	2	5	3	1.3	3.2	88.4	TS
26	0.4	40	5	2	3	1.5	2.9	89.8	TS
27	0.2	4	2	10	40	1.1	2.0	90.1	EI
28	0.1	40	5	1	5	1.0	1.9	88.1	TS
29	0.9	2	3	90	10	1.0	3.5	87.8	TS
30	0.2	4	1	10	40	2.6	3.1	90.1	EI
31	0.4	24	1	3	5	0.4	1.1	87.5	TS

Table S2 Dataset of the samples shown in Figure 3A

Number	Ratio	Layer repetition	Volume fraction	YBCO+BHO thickness (nm)	Pure thick-ness (nm)	J_c(20K, 9T) (MA/cm^2)
5	0.8	30	1.5	5.3	1.3	5.6
13	0.8	34	2	4.7	1.2	2.3
15	0.8	30	1	5.3	1.3	2.3
16	0.8	28	2	5.7	1.4	7.4
17	0.8	26	2	6.2	1.5	2.5
19	0.8	30	2	5.3	1.3	1.8
20	0.8	28	1.5	5.7	1.4	1.2
22	0.8	34	1.5	4.7	1.2	3.5
16-1	0.8	28	2	5.7	1.4	2.6
16-2	0.8	28	2	5.7	1.4	3.5
16-3	0.8	28	2	5.7	1.4	3.3
16-4	0.8	28	2	5.7	1.4	2.2
16-5	0.8	28	2	5.7	1.4	0.6
16-6	0.8	28	2	5.7	1.4	1.5
16-7	0.8	28	2	5.7	1.4	1.4
16-8	0.8	28	2	5.7	1.4	2.5

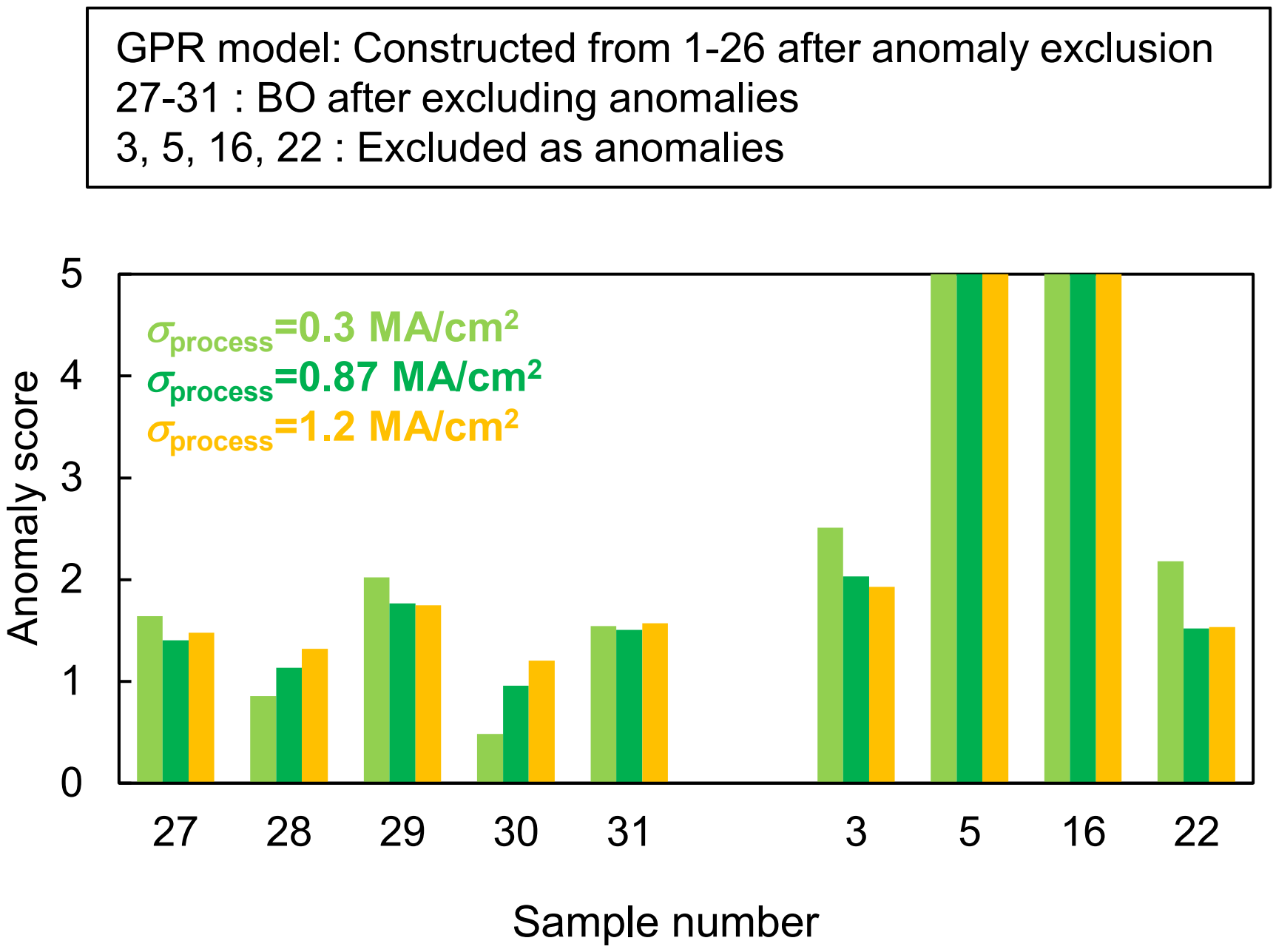

Figure S1 Anomaly score for each sample for different $\sigma_{process}$. The anomaly scores were evaluated for both the samples obtained by Bayesian optimization (BO) after anomaly exclusion (Samples 27-31) and the excluded anomalous samples (Samples 3, 5, 16, 22). The anomaly scores were calculated based on the GPR model constructed using Samples 1-26 after anomaly exclusion.

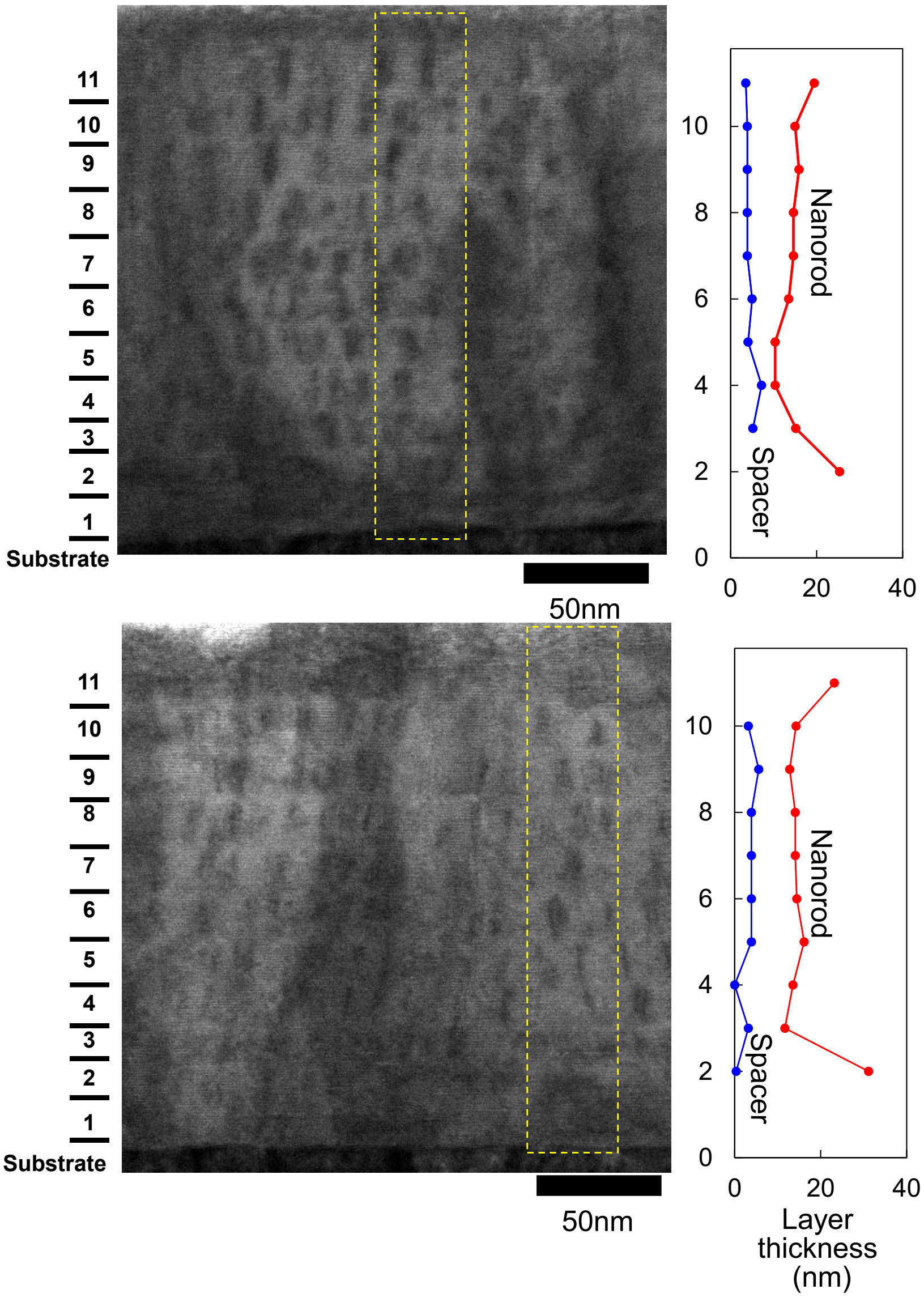

Figure S2 Cross-sectional STEM images of Sample 16 observed at different positions. The numbers indicate the layer indices. The right panels show the layer thickness as a function of position along the thickness direction, determined from the regions highlighted by yellow boxes in the left panels.

Table S3 Structural parameters of multilayers of Sample 16. These are determined from the STEM images shown in Figure S2

	Top panel in Figure S2	Bottom panel in Figure S2
Total thickness	195 nm	192 nm
Nanorod layer	14.3±2.6 nm	14.9±3.1 nm
Spacer layer	4.5±1.1 nm	3.4±1.5 nm
Nanorod layer ratio	76%	81%

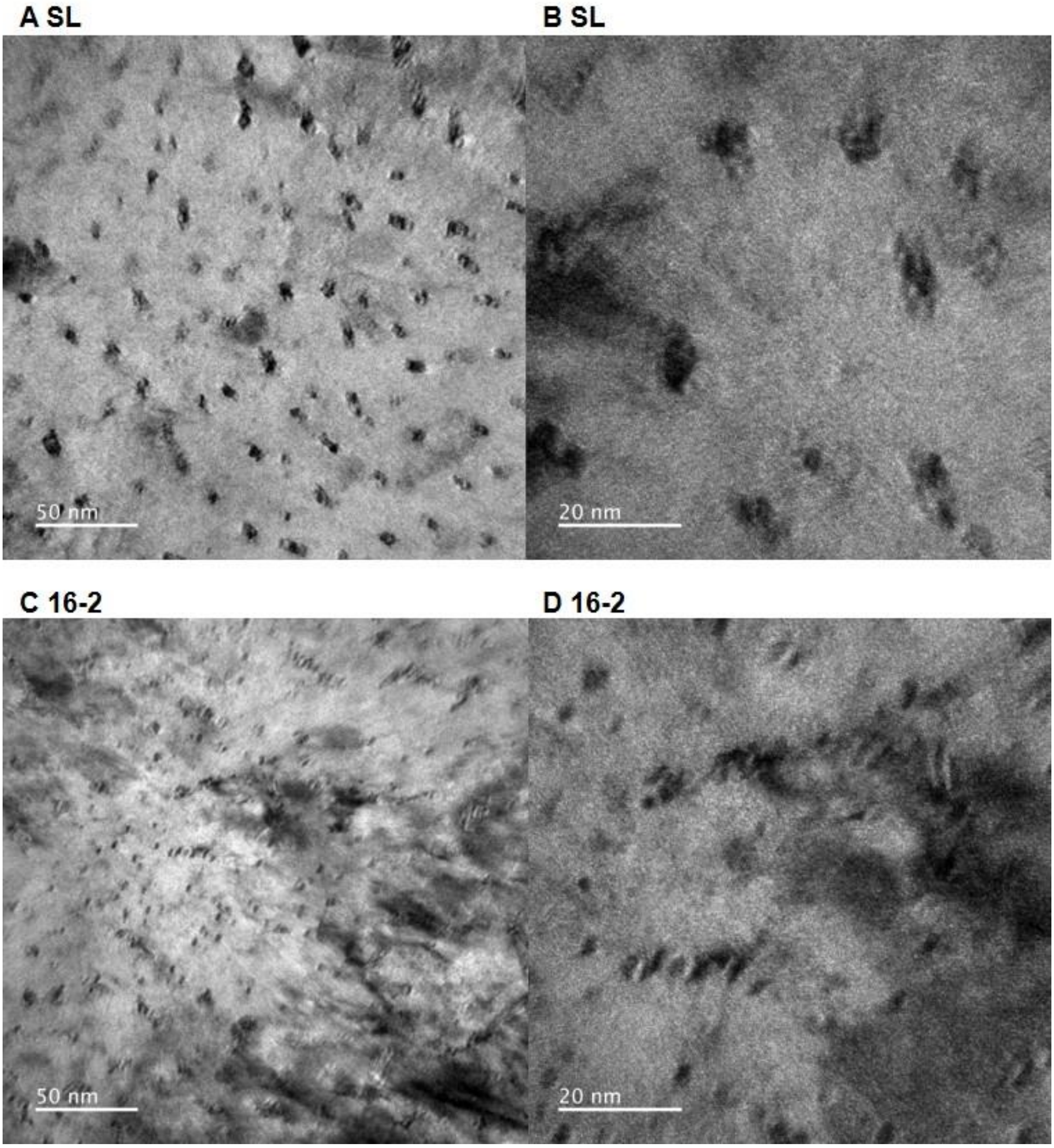

Figure S3 Plan-view TEM images of the SL(A, B) and Sample 16-2(C, D)

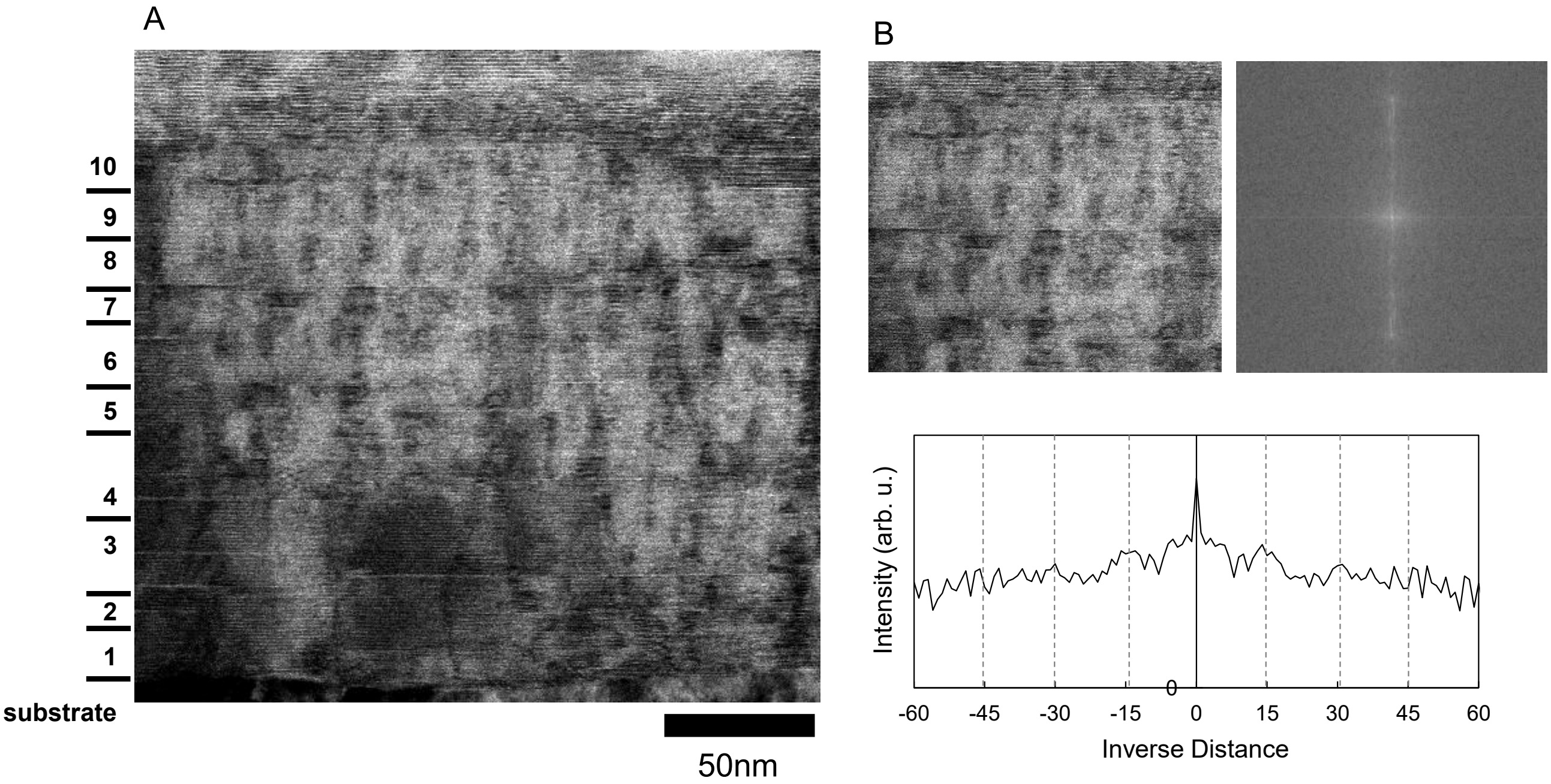

Figure S4 A. Cross-sectional STEM image of Sample 5. B. Cropped region from A (upper left), the corresponding FFT spectrum (upper right), and the vertical FFT profile including the origin (bottom).

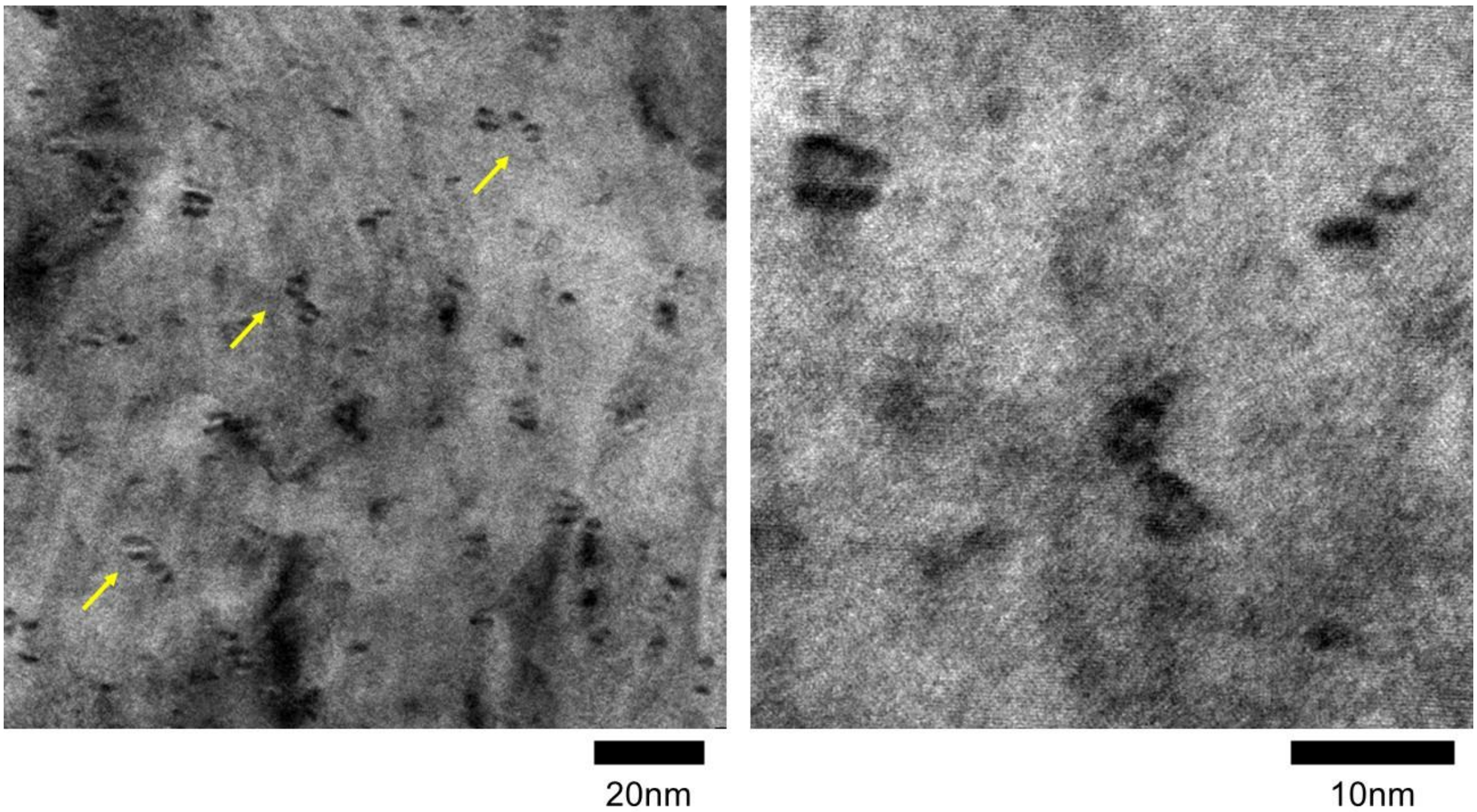

Figure S5. Plan-view STEM image of Sample 5. Arrows indicate two nanorods located in close proximity and partially overlapping.

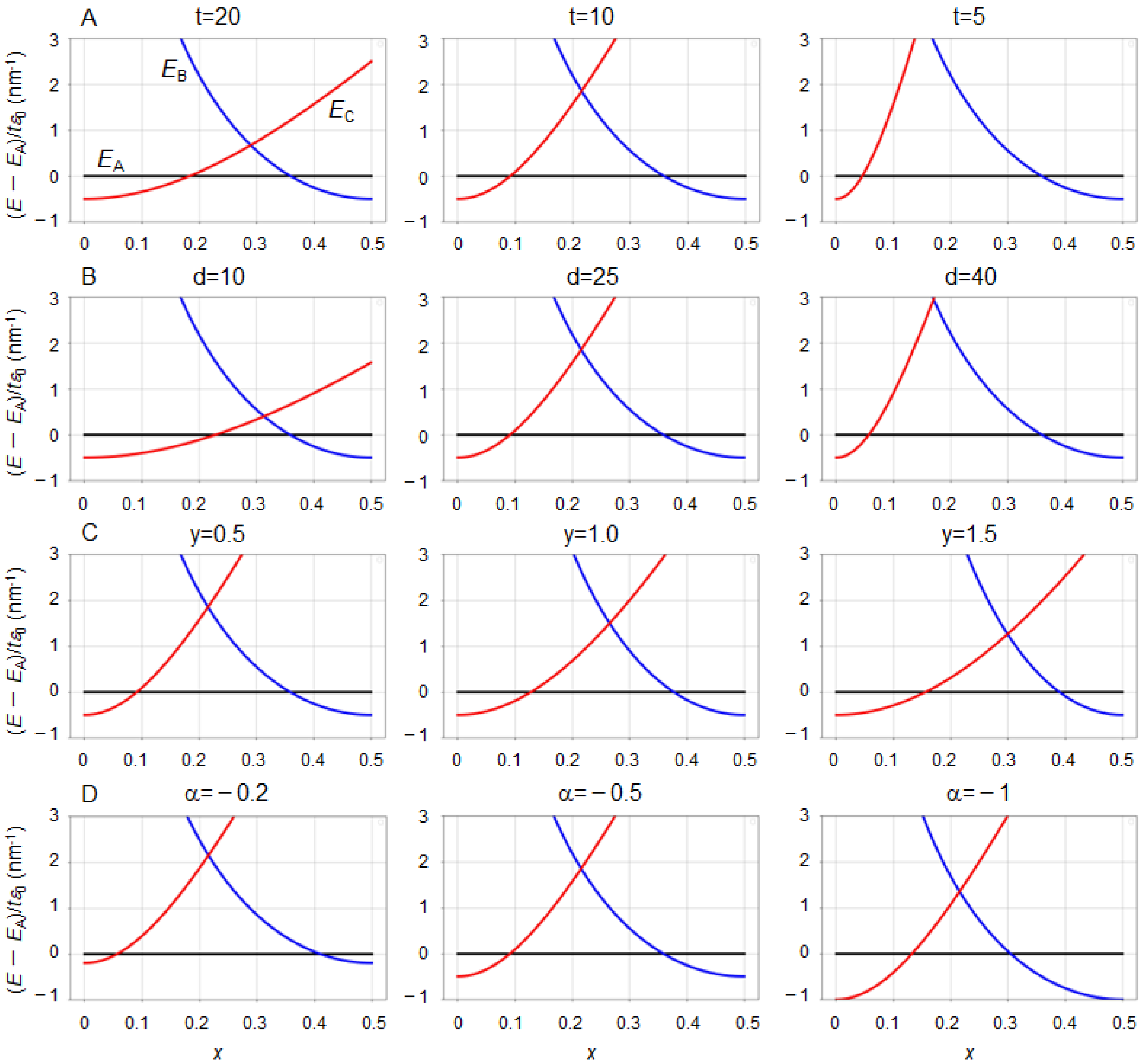

Figure S6 System energy as a function of nanorod misalignment, x for various parameters. A. Dependence of nanorod length (t) on system energy for $\alpha=-0.5$, $y=0.5$, $d=25$ nm ($d/\lambda=1/8$). B. Dependence of nanorod spacing (d) on system energy for $\alpha=-0.5$, $y=0.5$, $t=10$ nm. C. Dependence of spacer layer thickness (ty) on system energy for $\alpha=-0.5$, $d=25$ nm ($d/\lambda=1/8$) , $t=10$ nm. D. Dependence of pinning efficiency (α) on system energy for $y=0.5$, $d=25$ nm ($d/\lambda=1/8$) , $t=10$ nm.

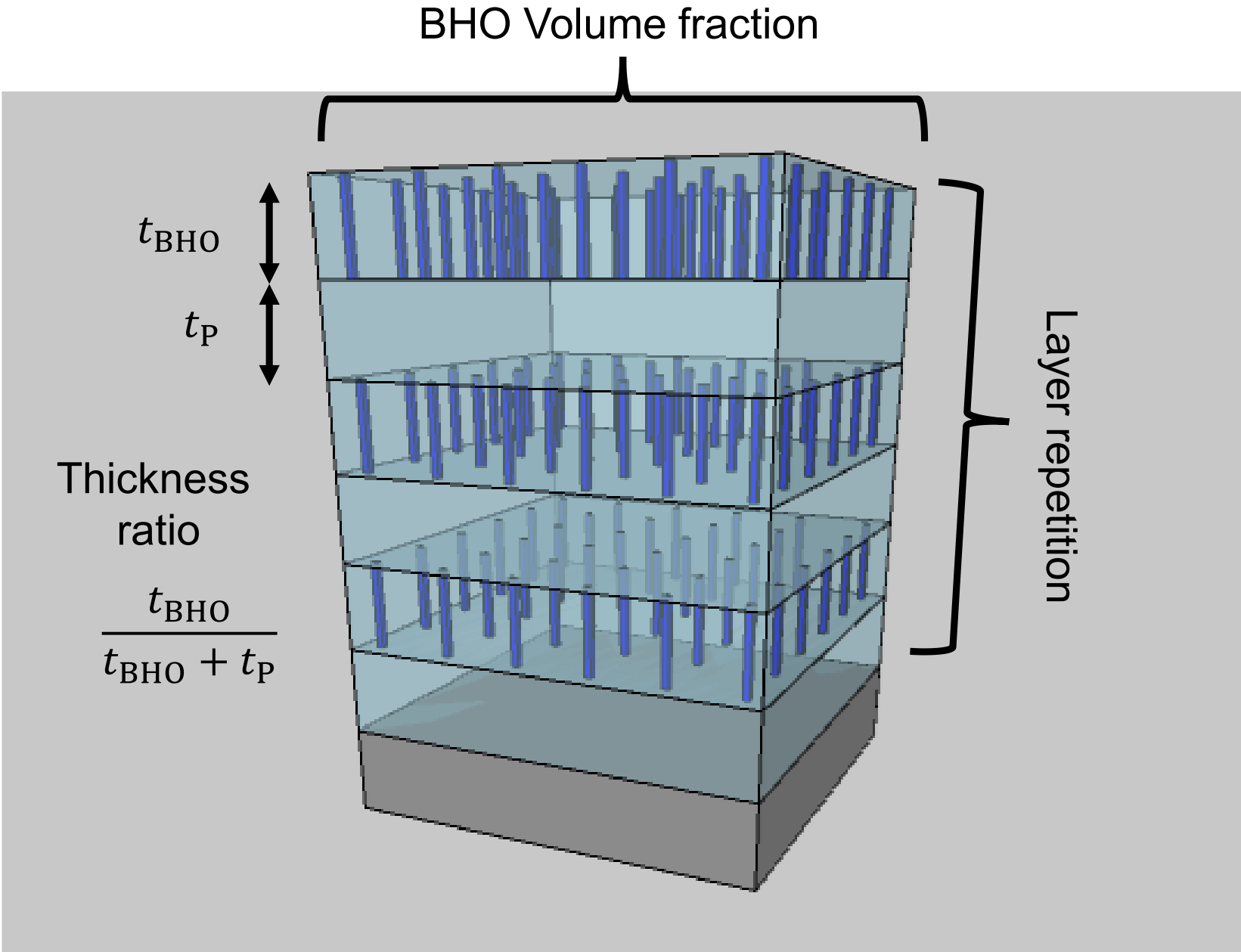

Figure S7 Schematic of the designed layer structure. While the structure may not always form as designed, the design parameters denote the sample specifications.
